# The TLX-miR-219 cascade regulates neural stem cell proliferation in neurodevelopment and schizophrenia iPSC model

**DOI:** 10.1038/ncomms10965

**Published:** 2016-03-11

**Authors:** Kiyohito Murai, Guoqiang Sun, Peng Ye, E. Tian, Su Yang, Qi Cui, Guihua Sun, Daniel Trinh, Olivia Sun, Teresa Hong, Zhexing Wen, Markus Kalkum, Arthur D. Riggs, Hongjun Song, Guo-li Ming, Yanhong Shi

**Affiliations:** 1 Division of Stem Cell Biology Research, Department of Developmental and Stem Cell Biology, Cancer Center, Beckman Research Institute of City of Hope, 1500 E. Duarte Road, Duarte, California 91010, USA; 2 Irell and Manella Graduate School of Biological Sciences, Beckman Research Institute of City of Hope, 1500 E. Duarte Road, Duarte, California 91010, USA; 3 Department of Diabetes and Metabolic Diseases Research, Beckman Research Institute of City of Hope, 1500 E. Duarte Road, Duarte, California 91010, USA; 4 Department of Molecular Immunology, Beckman Research Institute of City of Hope, 1500 E. Duarte Road, Duarte, California 91010, USA; 5 Institute for Cell Engineering, Johns Hopkins University School of Medicine, Baltimore, Maryland 21205, USA; 6 Department of Neurology, Johns Hopkins University School of Medicine, Baltimore, Maryland 21205, USA; 7 The Solomon Snyder Department of Neuroscience, Johns Hopkins University School of Medicine, Baltimore, Maryland 21205, USA; 8 Department of Psychiatry and Behavioral Science, Johns Hopkins University School of Medicine, Baltimore, Maryland 21205, USA

## Abstract

Dysregulated expression of miR-219, a brain-specific microRNA, has been observed in neurodevelopmental disorders, such as schizophrenia (SCZ). However, its role in normal mammalian neural stem cells (NSCs) and in SCZ pathogenesis remains unknown. We show here that the nuclear receptor TLX, an essential regulator of NSC proliferation and self-renewal, inhibits miR-219 processing. miR-219 suppresses mouse NSC proliferation downstream of TLX. Moreover, we demonstrate upregulation of miR-219 and downregulation of TLX expression in NSCs derived from SCZ patient iPSCs and *DISC1*-mutant isogenic iPSCs. SCZ NSCs exhibit reduced cell proliferation. Overexpression of TLX or inhibition of miR-219 action rescues the proliferative defect in SCZ NSCs. Therefore, this study uncovers an important role for TLX and miR-219 in both normal neurodevelopment and in SCZ patient iPSC-derived NSCs. Moreover, this study reveals an unexpected role for TLX in regulating microRNA processing, independent of its well-characterized role in transcriptional regulation.

## Abstract (web summary)

Dysregulation of microRNAs has been implicated in neurodevelopmental disorders, including schizophrenia. Here the authors show that the TLX-miR-219 cascade regulates the proliferation of neural stem cells during normal development, and this pathway is dysregulated in a schizophrenia iPSC model.

## Graphical abstract

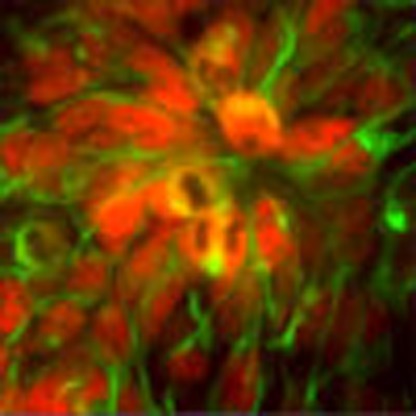

TLX is a nuclear receptor that plays a critical role in vertebrate brain function^1^,^2^,^3^. It is an essential regulator of adult neural stem cell (NSC) self-renewal^3^,^4^,^5^ and plays an important role in enhancing learning and memory by regulating adult hippocampal neurogenesis^6^,^7^. It also plays a role in neurodevelopment through regulation of cell cycle progression in embryonic NSCs^4^,^8^,^9^,^10^. TLX is a well-characterized transcriptional regulator. It controls target gene expression partly by recruiting transcriptional co-repressors, such as histone deacetylases (HDACs) and lysine-specific histone demethylase 1 (LSD1) (refs 11, 12, 13). TLX represses the transcription of GFAP, p21, pten and microRNAs (miRNAs), miR-9 and miR-137, but activates Wnt signalling, SIRT1 and MASH1 in NSCs^3^,^5^,^11^,^12^,^14^,^15^,^16^. However, the function of TLX in regulating gene expression beyond transcriptional regulation has not been reported.

miRNAs are small non-coding RNAs that regulate gene expression through translational inhibition or RNA degradation^17^. The biogenesis of miRNAs starts from primary transcripts (pri-miRNAs), which are processed by the nuclear RNase III Drosha into precursor miRNAs (pre-miRNAs) that contain hairpin loop structures. The pre-miRNAs are exported to the cytoplasm and further processed into mature miRNAs by the cytoplasmic RNase III Dicer. Mature miRNAs are incorporated into an RNA-induced silencing complex to repress target mRNAs. The Drosha complex consists of Drosha, DiGeorge syndrome critical gene 8 (DGCR8), RNA helicase p68 (DDX5) and p72 (DDX17)^18^,^19^,^20^. Recent studies reported that SMAD, p53 and BRCA1 bind to Drosha and promote the processing activity of Drosha^21^,^22^,^23^. However, our knowledge about the physiological effect of miRNA processing is still limited.

miR-219 is a miRNA that is specifically expressed in the brain^24^,^25^. It promotes oligodendrocyte differentiation by repressing negative regulators of oligodendrocyte differentiation^26^,^27^. In a recent study, miR-219 was shown to promote neural precursor cell differentiation in zebrafish by inhibiting apical polarity proteins, par-3 family cell polarity regulator (PARD) and protein kinase C iota (PRKCI)^28^. However, whether miR-219 regulates the phenotypes of neural stem/progenitor cells (collectively referred to as NSCs) in mammalian brains remains unknown.

Several studies analysing miRNA levels in post-mortem brains from schizophrenia (SCZ) patients reported an increase in the expression of a set of miRNAs in SCZ patients^29^,^30^,^31^. miR-219 is among the most highly upregulated miRNAs in brain regions of SCZ patients^29^,^30^,^32^. However, the functional relevance of elevated miR-219 expression in SCZ brain cells remains unknown.

In this study, we demonstrate a role for miR-219 in the regulation of mammalian NSC proliferation and differentiation, and identify TLX as an upstream regulator of miR-219. Moreover, we identify p68, Drosha and DGCR8 as novel TLX-interacting molecules, and uncover an unexpected role for TLX in regulating miRNA processing at the post-transcriptional level. Furthermore, we show that miR-219 expression is upregulated, whereas TLX expression is downregulated in SCZ NSCs. Overexpression of TLX or a miR-219 inhibitor is able to rescue the proliferative defects in SCZ NSCs.

## Results

### TLX represses miR-219 processing

**Figure 1:**
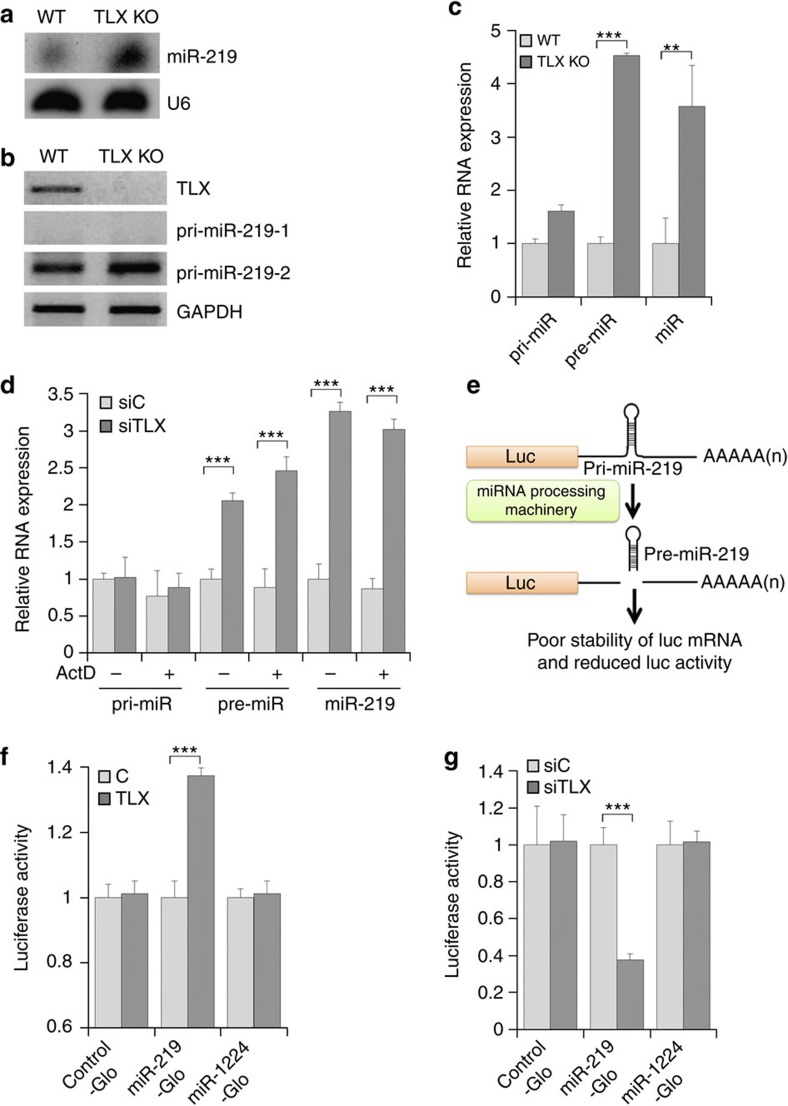
TLX inhibits miR-219 processing in NSCs. (**a**) Elevated expression of mature miR-219 in TLX KO mouse brains, compared to WT mouse brains, revealed by northern blot analysis. U6 is included as a loading control. (**b**) The levels of the two primary forms of miR-219, pri-miR-219-1 and pri-miR-219-2, exhibited minimal change in WT and TLX KO mouse brains, as analysed by RT–PCR. (**c**) The levels of pre-miR-219 and mature miR-219, but not pri-miR-219, increased significantly in TLX KO mouse brains; *n*=3. (**d**) The levels of pre-miR-219 and mature miR-219, but not pri-miR-219, increased significantly in TLX knockdown NSCs independent of actinomycin D treatment. siC: control RNA; siTLX: TLX siRNA; *n*=6. (**e**) A scheme for monitoring miRNA processing using a luciferase reporter. The miRNA processing activity is inversely correlated with the luciferase activity. (**f**) Overexpression of TLX inhibits miR-219 processing from pri-miR-219 to pre-miR-219. miR-1224-Glo was included as a negative control. The firefly luciferase activity was normalized with the *Renilla* luciferase internal control. The relative luciferase activity is shown. C: control vector; *n*=4. (**g**) Knockdown of TLX promotes miR-219 processing from pri-miR-219 to pre-miR-219. The relative luciferase activity in NSCs transfected with a vector expressing siC or siTLX, together with the control-Glo, miR-219-Glo reporter or miR-1224-Glo reporter control; *n*=4. **P<0.01 and ***P<0.001 by Student's *t*-test, and ‘n' represents experimental repeats in panels (**c**,**d**,**f**,**g**). Error bars are s.d. of the mean for all the quantification in this study. For each representative image, the experiments were repeated three times or more.

When comparing gene expression in wild-type (WT) and *TLX* knockout (KO) mouse brains, we detected markedly elevated expression of miR-219 in *TLX* KO brains (Fig. 1a). Because TLX is a transcription factor, we next examined the level of primary transcripts of *miR-219*. miR-219 is derived from two primary transcripts, pri-miR-219-1 and pri-miR-219-2. No expression of pri-miR-219-1 was detected in both WT and *TLX* KO brains, and not much change in the expression of pri-miR-219-2 was observed in WT and *TLX* KO brains either (Fig. 1b,c). Because we only detected pri-miR-219-2 in the brain, pri-miR-219-2 is referred to as pri-miR-219 hereafter.

We next determined the expression levels of the precursor form of miR-219 (pre-miR-219) in *TLX* KO brains. The level of pre-miR-219 increased substantially in *TLX* KO brains, compared to WT brains, similar to the change in mature miR-219 level, whereas no marked change was observed in pri-miR-219 level (Fig. 1c). We then examined the levels of all three forms of miR-219 in *TLX* knockdown NSCs. Knockdown of *TLX* by siRNA was confirmed by PCR with reverse transcription (RT–PCR; Supplementary Fig. 1). Consistent with our observation in *TLX* KO brains, considerable increase in the levels of pre-miR-219 and mature miR-219 was seen in *TLX* knockdown NSCs, compared to control NSCs, whereas minimal change was detected in the level of pri-miR-219 (Fig. 1d). The upregulation of pre-miR-219 and mature miR-219 by *TLX* knockdown was not affected by the treatment of the transcriptional inhibitor actinomycin D (Fig. 1d). These results suggest that TLX regulates the expression level of miR-219 at the post-transcriptional level, presumably through inhibiting the processing of miR-219 from the primary form to the precursor form.

To confirm that TLX plays a role in miR-219 processing, we performed a luciferase-based processing assay. HEK293T cells were transfected with a luciferase reporter construct containing pri-miR-219 sequences that include the Drosha/DGCR8-binding sites. The pri-miR-219 sequences were placed between the coding region of the luciferase gene and its polyadenylation signal. Cleavage of polyadenylation tails from the luciferase transcripts by Drosha/DGCR8 would induce degradation of the luciferase transcripts and reduce luciferase activity (Fig. 1e). We found that ectopic expression of *TLX* in HEK293T cells reduced miR-219 processing, as revealed by increased luciferase activity of miR-219-Glo (Fig. 1f). Expression of *TLX* had no effect on luciferase activity of miR-1224-Glo, a reporter that contains part of miR-1224, a miRtron that is processed into pre-miRNA independent of Drosha cleavage^33^ (Fig. 1f). In contrast to overexpression of *TLX*, knockdown of *TLX* in NSCs promoted miR-219 processing, as shown by reduced luciferase activity of miR-219-Glo, compared to control RNA-treated cells (Fig. 1g), but had no effect on luciferase activity of miR-1224-Glo (Fig. 1g). These results indicate that TLX negatively regulates miR-219 processing from the primary form to the precursor form.

### TLX interacts with the miRNA processing machinery

**Figure 2:**
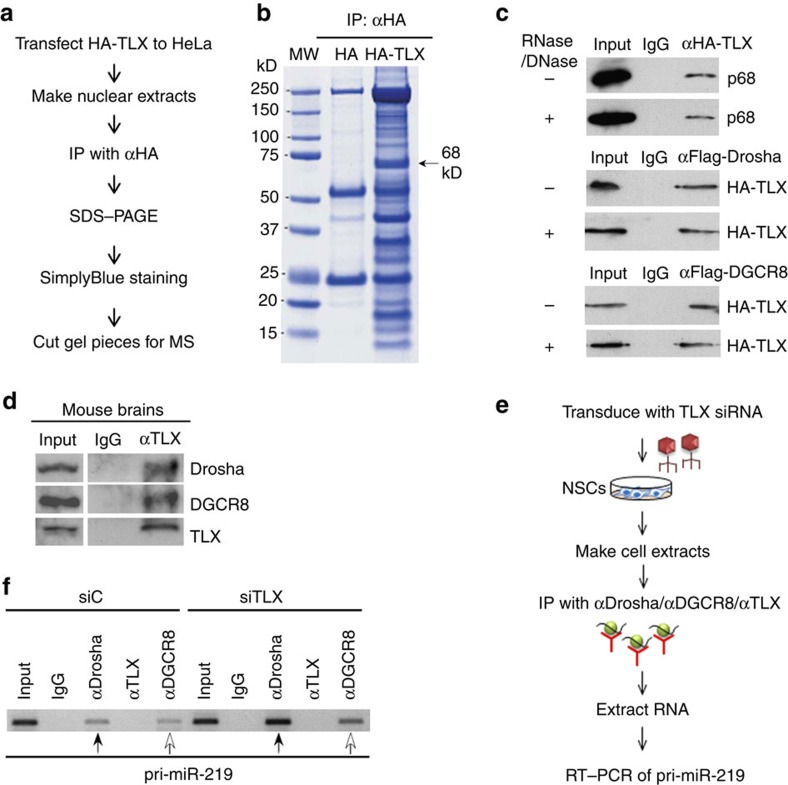
TLX interacts with the miRNA processing machinery. (**a**) A scheme for identifying TLX-interacting proteins using mass spectrometry (MS) analysis. (**b**) Differentially represented proteins in the HA immunoprecipitates of control HA or HA-TLX-expressing HeLa cells. Arrow indicates a protein band of 68 kD that is specifically detected in the HA immunoprecipitates of HA-TLX-expressing HeLa cells. (**c**) Interaction of TLX with p68, Drosha and DGCR8. Lysates of HA-TLX transfected HEK293T cells were treated with or without DNase and RNase, then immunoprecipitated with HA antibody or IgG control. The immunoprecipitates were blotted with p68 antibody. In parallel, lysates of Flag-Drosha and HA-TLX or Flag-DGCR8 and HA-TLX co-transfected HEK293T cells were treated with or without DNase and RNase. Cell lysates were immunoprecipitated with anti-Flag antibody, then blotted with anti-HA antibody. (**d**) Interaction of TLX with Drosha and DGCR8 in mouse brains. Lysates of embryonic mouse brains were immunoprecipitated with TLX antibody, then blotted with anti-Drosha, anti-DGCR8 or anti-TLX antibody. (**e**) A scheme for RNA immunoprecipitation. Lysates of NSCs transduced with TLX siRNA were immunoprecipitated with anti-Drosha, anti-DGCR8 or anti-TLX antibody. RNAs were extracted from the immunoprecipitates, and subjected to RT–PCR for pri-miR-219. (**f**) TLX knockdown promoted the binding of Drosha and DGCR8 to pri-miR-219. Lysates of NSCs transduced with siC or siTLX were immunoprecipitated with IgG control or indicated antibodies. pri-miR-219 RNA associated with Drosha (indicated by solid arrows) or DGCR8 (indicated by open arrows) was determined by RT–PCR.

In a parallel effort, we sought to identify novel TLX-interacting proteins. Nuclear extracts of HA-TLX-expressing HeLa cells were immunoprecipitated with an HA antibody. Proteins specifically pulled down in HA-TLX-expressing cells, but not in control cells, were subjected to mass spectrometry (MS) analysis to determine their identity (Fig. 2a,b). The RNA helicase p68 is among the proteins that were uniquely represented in the pull-downs of HA-TLX-expressing cells. Seventeen peptides of p68 were detected in the HA immunoprecipitates of HA-TLX-expressing cells, but not in that of control HA-expressing cells.

To confirm the interaction of TLX with p68, HEK293T cells were transfected with HA-TLX. p68 was detected in the HA-TLX immunocomplex and the interaction was not affected by the treatment with DNase and RNase (Fig. 2c). Because p68 is a component of the Drosha complex that processes pri-miRNAs into pre-miRNAs^18^,^19^, we hypothesized that TLX could interact with the miRNA processing machinery via its interaction with p68. To test whether TLX interacts with Drosha and DGCR8, HEK293T cells were transfected with Flag-Drosha or Flag-DGCR8 and HA-TLX. HA-TLX was detected in the immunocomplexes of both Flag-Drosha and Flag-DGCR8, independently of DNase and RNase treatment (Fig. 2c).

To confirm the interaction of endogenous TLX with Drosha/DGCR8, we collected E13.5 mouse brains, where TLX is highly expressed^4^. Brain lysates were immunoprecipitated with a TLX-specific antibody. Both Drosha and DGCR8 were detected in the TLX immunocomplex (Fig. 2d). These results indicate that TLX interacts with components of the miRNA processing machinery.

The interaction of TLX with Drosha and DGCR8 led us to hypothesize that TLX could inhibit miR-219 processing by preventing the miRNA processing machinery from binding to pri-miR-219. To test this hypothesis, we made cell lysates from NSCs transduced with lentivirus expressing TLX siRNA and performed RNA immunoprecipitation to determine if knockdown of TLX would affect the binding of Drosha and DGCR8 to pri-miR-219 (Fig. 2e). Weak binding of Drosha and DGCR8 to pri-miR-219 was detected in NSCs transduced with lentivirus expressing a control RNA, and no binding of TLX to pri-miR-219 was detected (Fig. 2f). Knockdown of TLX increased the binding of both Drosha and DGCR8 to pri-miR-219 substantially. These results indicated that TLX inhibits Drosha and DGCR8 from binding to pri-miR-219.

### miR-219 inhibits mammalian NSC proliferation

**Figure 3:**
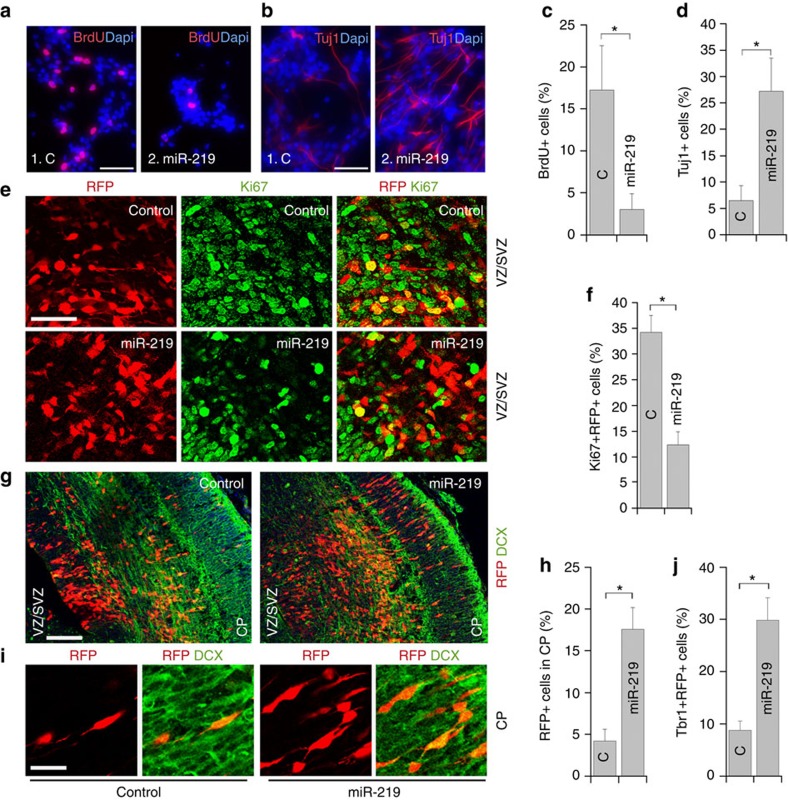
miR-219 inhibits NSC proliferation and promotes neuronal differentiation. (**a**,**b**) Overexpressing miR-219 in NSCs inhibited cell proliferation (**a**) and promoted neuronal differentiation (**b**). BrdU or Tuj1 staining is shown in red and Dapi counterstaining is shown in blue. (**c**,**d**) Quantification of BrdU+ cells (**c**) and Tuj1+ cells (**d**) in control RNA (C) and miR-219-treated NSCs. *n*=5; *P<0.001 by Student's *t*-test for both panels. ‘n' represents experimental repeats. (**e**) *In utero* electroporation of miR-219 decreased NSC proliferation in the VZ/SVZ of embryonic brains. Electroporated cells were labelled with RFP and proliferating cells were labelled with Ki67. (**f**) The percentage of RFP+Ki67+ cells out of total RFP+ cells in control RNA or miR-219-electroporated brains. *n*=3 mice per group. *P<0.01 by Student's *t*-test. (**g**) Electroporation of miR-219 induced precocious outward cell migration. The electroporated brains were stained for neuronal marker doublecortin (DCX). Transfected cells were labelled with RFP. (**h**) The percentage of electroporated cells (RFP+) that migrated to the CP. *n*=3 mice per group. *P<0.01 by Student's *t*-test. (**i**) Higher magnification images of RFP+DCX+ cells at the CP of brains electroporated with control RNA or miR-219. Scale bar, 50 μm for panels (**a**,**b**,**e**); 100 μm for panel (**g**); and 25 μm for panel (**i**). (**j**) The percentage of Tbr1+RFP+ cells out of total RFP+ cells in control RNA or miR-219-electroporated brains. *n*=3 mice per group. *P<0.01 by Student's *t*-test.

Because TLX plays an important role in regulating mammalian NSC proliferation and differentiation^3^,^4^, our observation that TLX regulates miR-219 processing in mouse NSCs led us to hypothesize that miR-219 could be involved in regulating mammalian NSC phenotypes. To test whether miR-219 affects mammalian NSC proliferation, NSCs were isolated from E14.5 mouse brains and treated with the miR-219 RNA duplex. BrdU labelling was performed to monitor cell proliferation. Treatment with miR-219 reduced cell proliferation substantially (Fig. 3a,c), but had minimal cytotoxicity (Supplementary Fig. 2a). These results indicate that miR-219 inhibits mammalian NSC proliferation. We next tested the effect of miR-219 on NSC differentiation. E14.5 mouse NSCs were treated with the miR-219 RNA duplex and cultured in differentiation medium. Treatment with miR-219 increased the percentage of βIII tubulin (Tuj1)-positive neurons substantially, compared to treatment with a control RNA (Fig. 3b,d). These results indicate that miR-219 promotes mammalian NSC differentiation into neurons.

To determine the effect of miR-219 on NSC regulation *in vivo*, miR-219 RNA duplex was electroporated together with an red fluorescent protein (RFP)-expressing vector into NSCs of E13.5 embryonic brains *in uterus*. The brains were dissected at E15.5 and analysed by immunohistochemistry. Immunostaining with Ki67, a proliferation marker, revealed that overexpression of miR-219 decreased cell proliferation in the ventricular zone and subventricular zone (VZ/SVZ) of mouse brains, where NSCs reside (Fig. 3e,f). To determine the effect of miR-219 overexpression on neuronal differentiation, immunostaining with doublecortin (DCX), a neuronal marker, was performed. Compared to control RNA-transfected cells, substantially more miR-219-electroporated (RFP+) cells migrated from VZ/SVZ to the cortical plate (CP), where neurons are located (Fig. 3g,h). Moreover, the RFP+ cells migrated to the CP were positive for DCX (Fig. 3i), confirming their neuronal identity. Immunostaining with Tbr1, another neuronal marker, was also performed. Quantification of the RFP+ cells that expressed Tbr1 revealed a much higher percentage of Tbr1+RFP+ cells in miR-219-electroporated brains than that in control RNA-electroporated brains (Fig. 3j). miR-219 has been shown to induce oligodendrocyte differentiation in *in utero* electroporated mouse brains collected at E17.5 (ref. 27). However, we did not detect the induction of either astrocyte marker GFAP or oligodendrocyte marker MBP expression in miR-219-electroporated brains collected at E15.5 (Supplementary Fig. 2b,c), presumably because we analysed brains at an earlier cortical developmental stage when neurogenesis is active but gliogenesis is not yet. These results indicate that miR-219 inhibits mammalian NSC proliferation and promotes their neuronal differentiation during early brain development.

### miR-219 acts downstream of TLX to regulate NSC phenotypes

**Figure 4:**
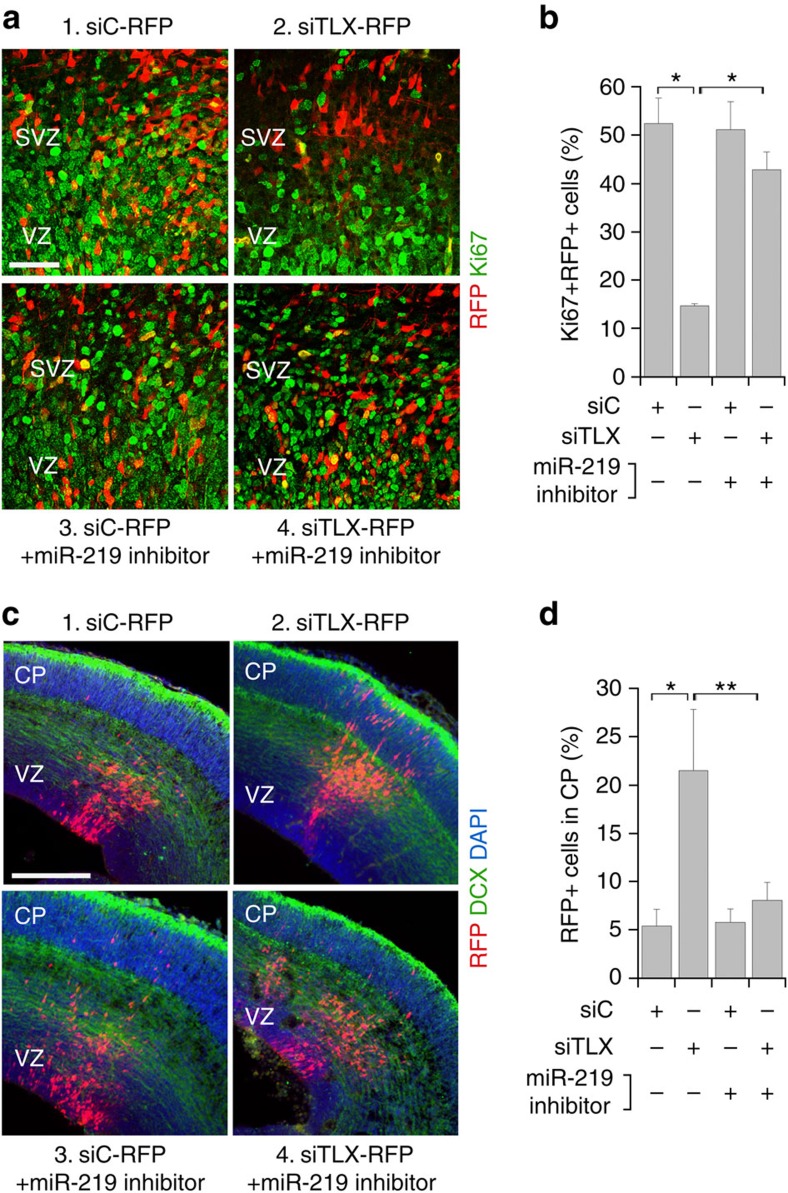
A miR-219 inhibitor reversed NSC phenotypes induced by TLX siRNA *in vivo*. (**a**) Co-electroporation of TLX siRNA with a miR-219 inhibitor rescued the decrease in NSC proliferation induced by TLX siRNA. E13.5 mouse brains were electroporated *in utero* with (1) a control RNA and the RFP reporter (siC-RFP); (2) TLX siRNA and the RFP reporter (siTLX-RFP); (3) a miR-219 inhibitor with siC-RFP; or (4) a miR-219 inhibitor with siTLX-RFP. The electroporated cells were labelled with RFP and proliferating cells were labelled with Ki67. (**b**) The percentage of RFP+Ki67+ cells out of total RFP+ cells in electroporated brains described in panel (**a**) is shown. *n*=3 mice per group. *P<0.001 by Student's *t*-test. (**c**) Electroporation was performed as described in panel (**a**) and brain sections were stained for neuronal marker DCX. Migration of the electroporated cells was tracked by RFP fluorescence. (**d**) The percentage of electroporated cells (RFP+) that migrated to the CP in electroporated brains described in panel (**c**) is shown. *n*=3 mice per group. *P<0.05 and **P<0.01 by Student's *t*-test. Scale bar, 50 μm for panel (**a**); 200 μm for panel (**c**).

To confirm that miR-219 acts downstream of TLX in NSCs, we tested whether the effect of *TLX* knockdown on NSC proliferation and differentiation could be rescued by a miRNA hairpin inhibitor that is designed specifically to inhibit miR-219 action by interfering with miR-219 binding to downstream targets. When TLX siRNA was electroporated into the VZ/SVZ of E13.5 mouse brains, the percentage of RFP+Ki67+ cells was decreased markedly, compared to that in control RNA-electroporated brains. However, when TLX siRNA and the miR-219 inhibitor RNA were co-electroporated, the percentage of RFP+Ki67+ cells was recovered substantially (Fig. 4a,b). On the other hand, an increasing number of TLX siRNA-electroporated cells migrated to the CP, compared to control cells (Fig. 4c,d). This phenotype is similar to the effect induced by electroporation of miR-219 into the VZ/SVZ of mouse brains (Fig. 3g,h). In brains co-electroporated with TLX siRNA and the miR-219 inhibitor, the percentage of transfected cells that migrated to the CP was also restored towards the control level (Fig. 4c,d). These results indicate that miR-219 is an important TLX downstream target in regulating mammalian NSC proliferation and differentiation *in vivo*.

### PDGFRα is a target gene of miR-219 and TLX in NSCs

To uncover mechanisms underlying miR-219-mediated regulation of NSC phenotypes, we identified potential miR-219 target genes using TargetScan, which revealed a set of candidate miR-219 tartgets, the 3′-untranslated region (3′-UTR) of which can base pair with miR-219. Among the candidate targets, *PDGFRα* is a confirmed miR-219 target^26^ and is expressed in NSCs^34^. RT–PCR showed that the expression of *PDGFRα* was markedly decreased in *TLX* KO brains (Supplementary Fig. 3a). Moreover, miR-219 repressed the activity of the luciferase reporter with the WT *PDGFRα* 3′-UTR, but not that with the mutant 3′-UTR, in which the base pairing with miR-219 was destroyed (Supplementary Fig. 3b), suggesting that miR-219 inhibits *PDGFRα* expression.

Moreover, the expression of *PDGFRα*, similar to *TLX*, was relatively high in NSCs but low in neurons, whereas the expression of *miR-219* was relatively low in NSCs but increased in neurons, inversely correlating to the expression of *PDGFRα* and *TLX* (Supplementary Fig. 3c–e), supporting the concept that TLX represses the expression of *miR-219*, while miR-219 inhibits the expression of *PDGFRα*.

Next we tested whether overexpressing *miR-219* or knockdown of *TLX* regulates *PDGFRα* expression. Repression of *PDGFRα* expression was detected in *miR-219*-transfected NSCs and TLX siRNA-transfected NSCs, respectively (Supplementary Fig. 3f), indicating that PDGFRα indeed acts downstream of miR-219 and TLX. To determine whether inhibition of *PDGFRα* by TLX is mediated by upregulating *miR-219*, we co-transduced NSCs with lentivirus expressing TLX siRNA and lentivirus expressing TuD-miR-219, a tough decoy inhibitor^35^ of miR-219. Co-expressing TuD-miR-219 with TLX siRNA rescued the inhibition of *PDGFRα* expression by TLX siRNA substantially (Supplementary Fig. 3g), suggesting that TLX regulates *PDGFRα* expression through miR-219. Taken together, these results demonstrate that *PDGFRα* is a downstream target gene of the TLX-miR-219 regulatory cascade.

We then tested how PDGFRα regulates NSC proliferation and differentiation *in vivo*. *In utero* electroporation of PDGFRα siRNA into E13.5 mouse brains reduced NSC proliferation as shown by decreased Ki67+RFP+ cells in the VZ/SVZ, whereas the number of RFP+ cells migrated to the CP was increased (Supplementary Fig. 4a–d). To determine whether PDGFRα is a critical downstream target gene of miR-219 in NSC regulation, we tested whether the effect of miR-219 on NSC proliferation and differentiation could be reversed by overexpressing *PDGFRα*. When PDGFRα and an RFP reporter were electroporated together with miR-219, the number of RFP+Ki67+ cells in the VZ/SVZ increased substantially (Supplementary Fig. 5a,c), whereas the RFP+ cells that migrated to the CP reduced considerably, compared to that in brains electroporated with RFP and miR-219 only (Supplementary Fig. 5b,d). These results indicate that *PDGFRα* is an important downstream target of miR-219 in regulating NSC proliferation and differentiation.

Because the expression of *PDGFRα* was reduced in *TLX* knockdown NSCs (Supplementary Fig. 3f,g), we determined whether overexpression of *PDGFRα* could rescue TLX siRNA-induced inhibition of NSC proliferation. Co-electroporation of PDGFRα with TLX siRNA to E13.5 mouse brains showed an increase in the number of Ki67+RFP+ cells in the VZ/SVZ and a decrease in the number of RFP+ cells migrated to the CP, compared to electroporation with TLX siRNA alone (Supplementary Fig. 6a–d). These results indicate that PDGFRα acts downstream of TLX to regulate NSC proliferation and differentiation in mammalian brains.

### A TLX peptide promotes miR-219 processing

**Figure 5:**
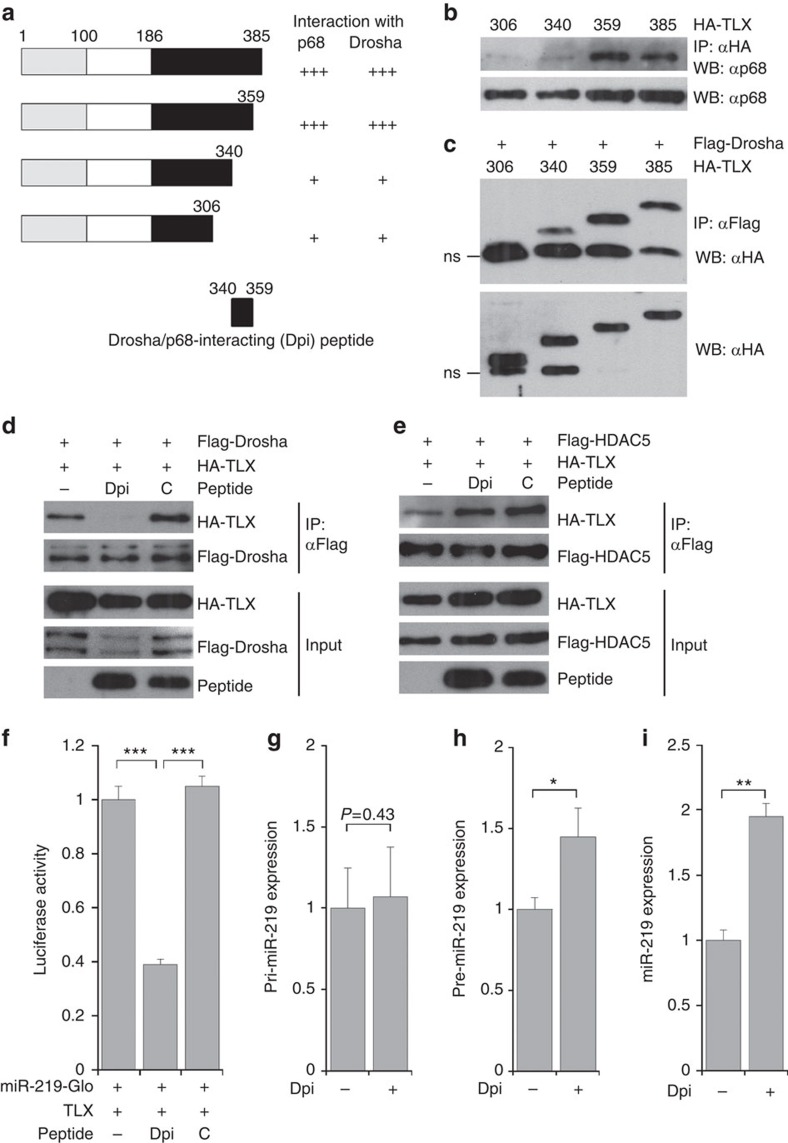
A TLX peptide promotes miR-219 processing. (**a**) Mapping p68 and Drosha-interacting domain in TLX. A schematic of TLX deletion mutants and the Drosha/p68-interacting domain (Dpi) is shown on the left. A summary of p68 and Drosha-binding results is shown on the right. (**b**) Deletion of TLX residues 340–359 reduced the interaction of TLX with p68 substantially. HEK293T cells were transfected with HA-tagged full-length TLX (residues 1–385) or its deletion mutants (residues 1–306, 1–340 or 1–359). Lysates were immunoprecipitated (IP) with HA antibody (αHA), then probed with p68 antibody (αp68) in western blot analysis (WB). (**c**) Deletion of TLX residues 340–359 reduced TLX interaction with Drosha. HEK293T cells were transfected with Flag-tagged Drosha and HA-tagged full length or deletion mutants of TLX. Lysates were IP with Flag antibody (αFlag), then probed with HA antibody (αHA). A nonspecific (ns) band in the western blot was indicated. (**d**,**e**) Expressing the Dpi peptide abolished the interaction of TLX with Drosha (**d**), but not the interaction of TLX with HDAC5 (**e**), as revealed by co-IP analysis. An empty vector (−) and a control peptide (C) were included as negative controls for the Dpi peptide. Cell lysates were IP with anti-Flag antibody, then blotted with anti-HA or anti-Flag antibody. The expression of individual proteins in the transfected cells was shown by immunoblotting as input. (**f**) Expression of the Dpi peptide promotes miR-219 processing. miR-219 processing was monitored using the miR-219-Glo reporter. Expressing the Dpi peptide decreased miR-219-Glo activity compared to expressing the empty vector (−) or a control peptide (C); *n*=3. ***P<0.001 by Student's *t*-test. (**g**–**i**) The levels of pre-miR-219 (**h**) and mature miR-219 (**i**), but not pri-miR-219 (**g**), were increased by expressing the Dpi peptide, as revealed by RT–PCR. *n*=4 (**g**); *n*=4 (**h**); *n*=3 (**i**). *P<0.05 and **P<0.01 by Student's *t*-test in panels (**h**,**i**). ‘n' represents experimental repeats in panels (**f**–**i**).

To determine the region in TLX that is critical for the interaction with the miRNA processing machinery, we mapped the minimal domain of TLX for p68 and Drosha interactions by serial deletion (Fig. 5a). Co-immunoprecipitation analysis revealed that the TLX residues 340–359 were critical for the TLX-p68 interaction (Fig. 5a,b). Deletion of TLX residues 340–359 also reduced the interaction of TLX with Drosha markedly (Fig. 5a,c). We therefore termed the TLX region spanning residues 340–359 as the Drosha/p68-interacting (Dpi) domain (Fig. 5a).

To determine whether the Dpi domain interferes with the TLX-Drosha interaction, an HA-tagged TLX peptide containing the Dpi domain (Dpi) was co-expressed with HA-tagged full-length TLX (HA-TLX) and Flag-Drosha in HEK293T cells. The interaction of TLX with Drosha, as determined by co-immunoprecipitation, was substantially reduced when Dpi was co-expressed, compared to that when an empty vector (−) or a control peptide (C) was expressed (Fig. 5d). To determine the specificity of Dpi on the interaction of TLX with its interacting partners, we expressed Dpi together with HA-TLX and Flag-HDAC5, a known transcriptional co-repressor of TLX^11^. In contrast to the interaction of TLX with Drosha, the interaction of TLX with HDAC5 was not blocked by Dpi (Fig. 5e). These results indicate that Dpi specifically interferes with the interaction of TLX with Drosha, but not the interaction of TLX with a transcriptional co-regulator.

To determine whether the interaction between TLX and the miRNA processing machinery is critical for regulation of miR-219 processing by TLX, we used Dpi to block the interaction of TLX with the miRNA processing machinery and miR-219-Glo, a luciferase reporter containing the pri-miR-219 sequence in its 3′-UTR to monitor miR-219 processing. Co-transfection of TLX with Dpi reduced the luciferase activity substantially, compared to transfection with TLX alone (Fig. 5f), suggesting that expression of Dpi promoted miR-219 processing from pri-miR-219. In contrast, a control peptide that contains TLX residues outside the Dpi domain failed to boost miR-219 processing (Fig. 5f).

We next electroporated the Dpi peptide into NSCs and determined miR-219 processing by evaluating the levels of the three forms of miR-219, pri-miR-219, pre-miR-219 and mature miR-219. The levels of both pre-miR-219 and mature miR-219 forms increased considerably, whereas no significant change was detected in the level of pri-miR-219 (Fig. 5g–i). These results further demonstrate that expressing the Dpi peptide promotes miR-219 processing but has no effect on the transcription of pri-miR-219.

**Figure 6:**
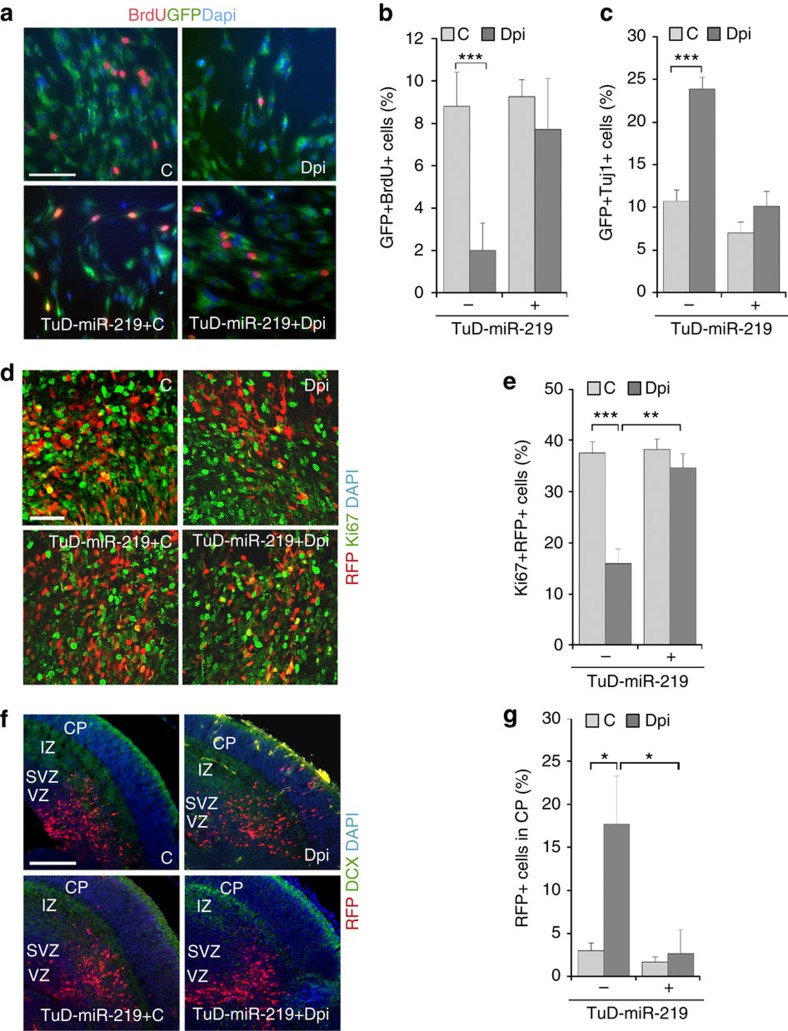
The Dpi peptide regulates NSC proliferation and differentiation. (**a**–**c**) Expression of the Dpi peptide inhibits NSC proliferation and promotes neuronal differentiation, and this effect could be reversed by the miR-219 inhibitor, TuD-miR-219. Mouse embryonic NSCs were transduced with virus expressing the Dpi peptide or a control peptide (C), in the absence or presence of TuD-miR-219. The virus-transduced cells were labelled with a GFP reporter. Cell proliferation was determined by the percentage of GFP+BrdU+ cells (BrdU+GFP+/GFP+; **b**) and neuronal differentiation was determined by the percentage of GFP+Tuj1+ cells (Tuj1+GFP+/GFP+; **c**). *n*=7 (**b**); *n*=5 (**c**). ‘n' represents experimental repeats. (**d**–**g**) Expression of Dpi inhibited NSC proliferation (**d**,**e**), but promoted neuronal differentiation (**f**,**g**) in mouse brains. E13.5 mouse brains were electroporated *in utero* with vectors expressing: (1) a control peptide and RFP reporter (C); (2) Dpi peptide and RFP reporter (Dpi); (3) TuD-miR-219 plus control peptide and RFP reporter (TuD-miR-219+C); or (4) TuD-miR-219 plus Dpi and RFP reporter (TuD-miR-219+Dpi). The electroporated cells were labelled with RFP, proliferating cells were labelled with Ki67 (**e**) and neuronal cells were labelled with DCX (**f**). The percentage of RFP+Ki67+ cells (**e**) or RFP+ cells that migrated to the CP (**g**) out of total RFP+ cells is shown. *n*=3 mice per group for panels (**e**,**g**). *P<0.05, **P<0.01 and ***P<0.001 by Student's *t*-test for panels (**b**,**c**,**e**,**g**). Scale bar, 100 μm for panel (**a**); 50 μm for panel (**d**); and 200 μm for panel (**f**).

Next we tested whether expressing Dpi could affect NSC proliferation and differentiation. NSCs from E14.5 mouse brains were transduced with lentivirus expressing Dpi or a control peptide and a green fluorescent protein (GFP) reporter. Compared to the control peptide, expression of Dpi reduced cell proliferation substantially (Fig. 6a,b). To determine whether inhibition of NSC proliferation by Dpi is mediated by modulating miR-219, we co-transduced NSCs with Dpi and the miR-219 decoy inhibitor, TuD-miR-219. Expressing TuD-miR-219 rescued the Dpi-mediated inhibition of NSC proliferation substantially (Fig. 6a,b), suggesting that Dpi regulates NSC proliferation by modulating miR-219. Treatment with Dpi also increased the percentage of Tuj1-positive neurons significantly, compared to treatment with the control peptide, and co-expressing TuD-miR-219 reversed this effect largely (Fig. 6c). These results together indicate that the Dpi peptide inhibits NSC proliferation and promotes neuronal differentiation by modulating miR-219 expression.

To determine the effect of Dpi on NSC proliferation and differentiation *in vivo*, we electroporated a vector expressing a control peptide or Dpi together with an RFP reporter into E13.5 mouse brains *in uterus*. Expression of Dpi decreased Ki67+RFP+ cells in the VZ/SVZ, compared to expression of the control peptide. Co-electroporating Dpi with TuD-miR-219 rescued the reduced cell proliferation induced by Dpi (Fig. 6d,e). These results indicate that Dpi inhibits NSC proliferation *in vivo*, presumably through regulating miR-219 expression. On the other hand, more RFP+ cells that had been electroporated with the Dpi-expressing vector migrated from the VZ/SVZ to the CP. Co-electroporating Dpi- with TuD-miR-219 reversed the precocious migration largely (Fig. 6f,g). These results indicate that Dpi promotes neuronal differentiation *in vivo* through modulating miR-219 action. Together with the observation that Dpi promotes miR-219 processing (Fig. 5f–i), these results suggest that modulation of miR-219 processing by TLX regulates NSC proliferation and differentiation.

### Elevated miR-219 expression inhibits SCZ NSC proliferation

**Figure 7:**
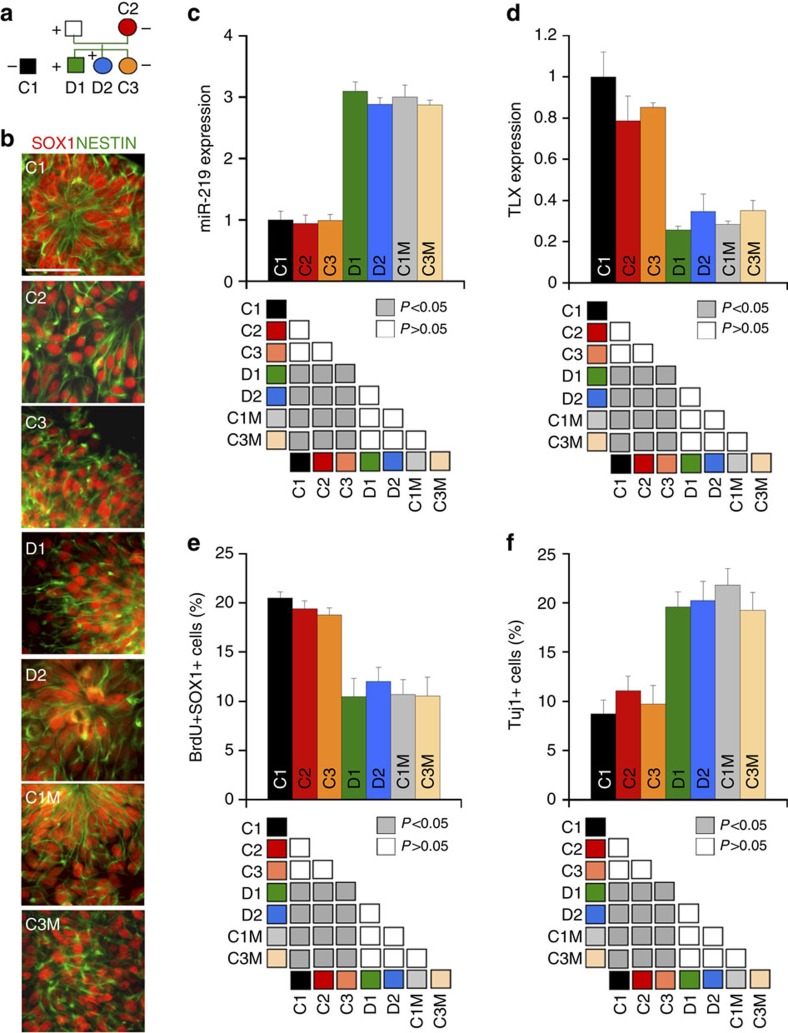
The DISC1-mutant NSCs exhibit increased miR-219 expression and reduced proliferation. (**a**) A schematic diagram showing the pedigree for iPSC generation. iPSCs from a wild-type (WT) individual outside of the pedigree (C1) were included as a control. The + and − signs represent the presence and absence of the 4-bp deletion in the DISC1 gene, respectively. The squares represent male, while the circles represent female. (**b**) NSCs derived from both WT (C1, C2 and C3) and DISC1-mutant iPSCs (D1, D2, C1M and C3M) expressed neural precursor markers, SOX1 and NESTIN. Scale bar, 50 μm. (**c**,**d**) RT–PCR showing elevated expression of miR-219 (**c**) and reduced expression of TLX (**d**) in DISC-mutant NSCs (D1, D2, C1M and C3M), compared to that in WT NSCs (C1, C2 and C3). (**e**,**f**) The DISC1-mutant NSCs (D1, D2, C1M and C3M) exhibited reduced cell proliferation (**e**) and precocious neuronal differentiation (**f**). NSC proliferation rate was determined by the percentage of BrdU+SOX1+ cells. Neuronal differentiation rate was determined by the percentage of Tuj1+ cells. *n*=4 for panels (**c**–**f**). ‘n' represents experimental repeats. ANOVA test result was shown below each graph.

Elevated expression of *miR-219* has been observed in various brain regions of SCZ patients^29^,^30^,^32^. The expression of *miR-219* in mammalian NSCs prompted us to ask whether *miR-219* expression is altered in SCZ NSCs. To address this question, we obtained induced pluripotent stem cells (iPSCs) derived from SCZ patients (D1 and D2; Fig. 7a) of pedigree H^36^ with a 4-bp deletion in the coding sequence of the *DISC1* gene^37^,^38^. This deletion causes a frameshift and premature termination of translation in DISC1 (refs 37, 38). We differentiated the SCZ iPSCs into NSCs. WT NSCs derived from iPSCs of unaffected individuals (C1, C2 and C3) were used as controls^37^,^38^. Both SCZ and WT iPSC-derived NSCs expressed human NSC markers SOX1 and NESTIN (Fig. 7b).

RT–PCR revealed that the level of miR-219 increased substantially in *DISC1*-mutant SCZ NSCs, compared to WT control NSCs (Fig. 7c). To determine whether the *DISC1* mutation is sufficient to induce elevated miR-219 expression in NSCs, we took advantage of the isogenic iPSC lines C1M and C3M, in which the 4-bp deletion seen in the SCZ patients was introduced into the *DISC1* gene in the WT control iPSC lines C1 and C3 (ref. 38). Similar to what was seen in the SCZ NSCs, we observed a considerable increase in miR-219 level in C1M and C3M NSCs, compared to that in their isogenic WT controls (Fig. 7c). These results together indicate that miR-219 expression is elevated in NSCs of *DISC1*-mutant SCZ patients, and that a *DISC1* mutation is sufficient to induce miR-219 upregulation in NSCs. In contrast to elevated *miR-219* expression, we observed reduced expression of *TLX* in both the *DISC1*-mutant SCZ NSCs (D1 and D2) and the genetically engineered C1M and C3M NSCs that contain the *DISC1* mutation (Fig. 7d). The inverse correlation between TLX and miR-219 expression in SCZ NSCs further supports our hypothesis that TLX negatively regulates miR-219 level in NSCs.

Reduced NSC proliferation has been observed in post-mortem brain specimens from SCZ patients^39^ and DISC1 has been shown to regulate NSC proliferation in the developing mouse cortex^40^,^41^. However, whether DISC1 regulates cell proliferation in human NSCs and whether mutant DISC1 induces abnormal NSC proliferation remained unknown. Our results showing that mutant DISC1 induces elevated expression of miR-219 in SCZ NSCs led us to hypothesize that increased expression of miR-219 induced by mutant DISC1 could result in abnormal cell proliferation in SCZ NSCs. To test this hypothesis, we first compared cell proliferation in WT and *DISC1*-mutant SCZ NSCs by BrdU and SOX1 double labelling. The percentage of BrdU+SOX1+ cells was significantly reduced in *DISC1*-mutant SCZ NSCs, compared to that in WT control NSCs (Fig. 7e). To test whether the *DISC1* mutation is sufficient for the observed proliferative defects, we compared cell proliferation in NSCs derived from the isogenic iPSC lines C1, C1M and C3, C3M. The decrease in NSC proliferation observed in SCZ NSCs was recapitulated in C1M and C3M NSCs (Fig. 7e). To determine whether abnormal expression of miR-219 in *DISC1*-mutant NSCs tips the balance between human NSC proliferation and differentiation, we compared neuronal differentiation in WT and *DISC1*-mutant NSCs under a spontaneous differentiation condition. Quantification of Tuj1+ neuronal cells revealed increased neuronal differentiation in *DISC1*-mutant NSCs, compared to WT NSCs (Fig. 7f), consistent with our observation of reduced NSC proliferation in *DISC1*-mutant NSCs (Fig. 7e).

**Figure 8:**
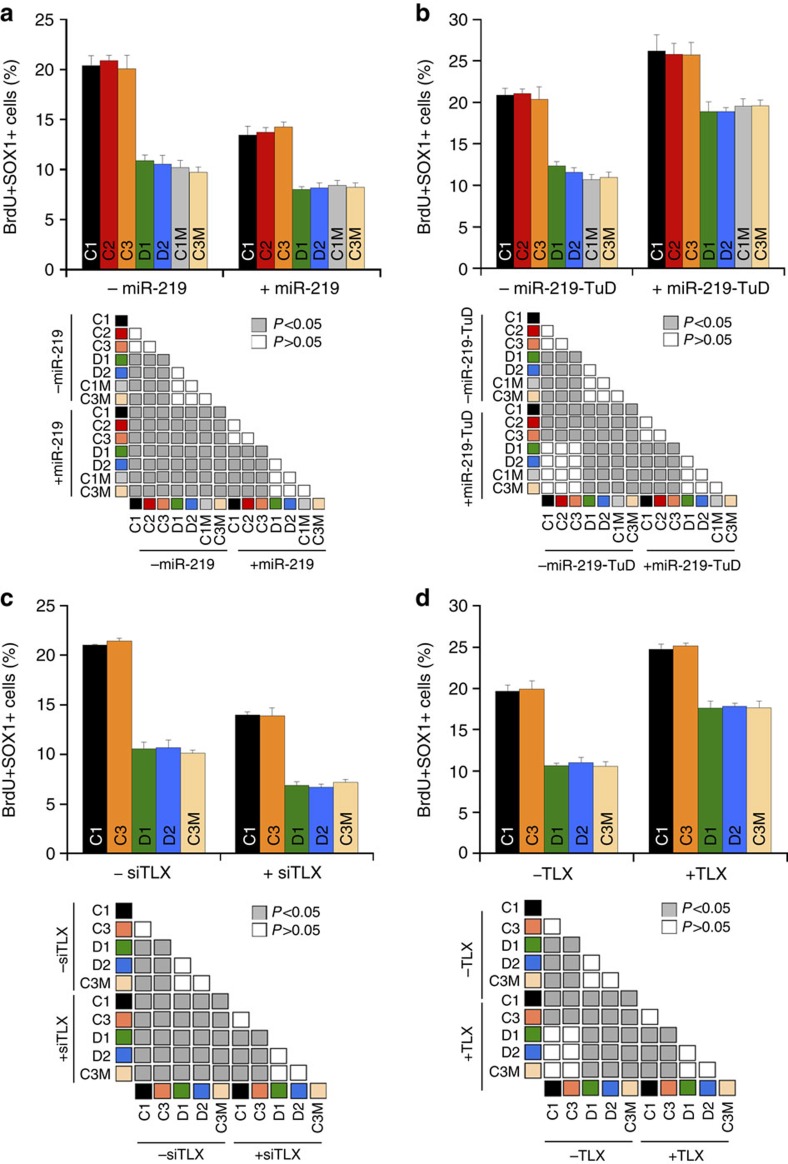
Inhibition of miR-219 or overexpression of TLX rescues reduced cell proliferation in SCZ NSCs. (**a**) Overexpression of miR-219 inhibited cell proliferation in WT NSCs. WT (C1, C2 and C3) and DISC1-mutant NSCs (D1, D2, C1M and C3M) were transduced with virus expressing a control vector (−miR-219) or miR-219-expresing vector (+miR-219). NSC proliferation rate was determined by the percentage of BrdU+SOX1+ cells. (**b**) TuD-miR-219 rescued the proliferative defect in DISC1-mutant NSCs. WT and DISC1-mutant NSCs were transduced with a control vector (−miR-219-TuD) or TuD-miR-219-expressing vector (+miR-219-TuD). NSC proliferation rate was determined by the percentage of BrdU+SOX1+ cells. (**c**) Knockdown of TLX inhibited cell proliferation in WT NSCs. WT (C1 and C3) and DISC1-mutant NSCs (D1, D2 and C3M) were transduced with virus expressing a control RNA (−siTLX) or TLX siRNA (+siTLX). NSC proliferation rate was determined by the percentage of BrdU+SOX1+ cells. (**d**) Overexpression of TLX rescued the proliferative defect in DISC1-mutant NSCs. WT and DISC1-mutant NSCs were transduced with control vector (−TLX) or TLX-expressing vector (+TLX). NSC proliferation rate was determined by the percentage of BrdU+SOX1+ cells. *n*=5 for panels (**a**,**b**) and *n*=4 for panels (**c**,**d**). ‘n' represents experimental repeats. ANOVA test result was shown below each graph.

To determine whether elevated miR-219 expression is sufficient to reduce cell proliferation in human NSCs, we overexpressed miR-219 in WT NSCs using a miR-219-expressing retroviral vector, and NSC proliferation was determined by BrdU and SOX1 double labelling. Reduced cell proliferation was observed in miR-219-overexpressing WT NSCs compared to control vector-expressing WT NSCs, in a manner similar to the reduced cell proliferation observed in *DISC1*-mutant NSCs when compared to WT NSCs (Fig. 8a). In parallel, the miR-219-overexpressing WT NSCs were induced for neuronal differentiation. The rate of neuronal differentiation was determined by the percentage of Tuj1+ cells. Overexpression of miR-219 in WT NSCs also increased neuronal differentiation rate, compared to that in control vector-treated WT NSCs (Supplementary Fig. 7a). These results indicate that elevated miR-219 expression is sufficient to inhibit cell proliferation in human NSCs. Together with our observation that miR-219 expression is abnormally upregulated in SCZ NSCs, these results suggest that the abnormally elevated miR-219 expression could be an underlying factor for reduced NSC proliferation observed in SCZ patients.

To determine whether the proliferative defect in *DISC1*-mutant NSCs indeed resulted from abnormally elevated miR-219 expression, we inhibited miR-219 in *DISC1*-mutant NSCs using TuD-miR-219. NSC proliferation was monitored by BrdU and SOX1 double labelling. Treating the *DISC1*-mutant NSCs (D1, D2, C1M and C3M) with TuD-miR-219 increased the proliferative rate in these cells substantially, largely rescuing the proliferative defects of the *DISC1*-mutant NSCs (Fig. 8b). Treatment with TuD-miR-219 also reversed the elevated differentiation in *DISC1*-mutant NSCs considerably, as revealed by the reduced percentage of Tuj1+ cells, compared to that in cells treated with a control vector (Supplementary Fig. 7b). These results indicate that miR-219 plays an important role in regulating cell proliferation in *DISC1*-mutant SCZ NSCs and that a miR-219 inhibitor could rescue the proliferative defect in these cells.

In addition to elevated *miR-219* expression in *DISC1*-mutant SCZ NSCs, we also detected reduced expression of *TLX* in *DISC1*-mutant SCZ NSCs (Fig. 7). To test whether *TLX* knockdown is able to reduce cell proliferation in human NSCs, we transduced WT NSCs with a TLX siRNA-expressing lentiviral vector. Reduced cell proliferation was observed in *TLX* knockdown WT NSCs, compared to control vector-transduced WT NSCs (Fig. 8c). To test whether increase of *TLX* expression could rescue the proliferative defect in SCZ NSCs, we overexpressed *TLX* in *DISC1*-mutant NSCs. Elevated expression of *TLX* in *DISC1*-mutant NSCs increased cell proliferation (Fig. 8d), consistent with the observation in *DISC1*-mutant NSCs treated with TuD-miR-219 (Fig. 8b). These results together indicate that the TLX-miR-219 cascade is important in regulating cell proliferation in *DISC1*-mutant SCZ NSCs.

## Discussion

In this study, we have demonstrated that TLX regulates miRNA processing independent of its well-characterized role in transcriptional regulation, and that miR-219 acts downstream of TLX to regulate NSC proliferation and differentiation in mammalian brains. Moreover, we have shown that *miR-219* expression is elevated, whereas *TLX* expression is reduced, in *DISC1*-mutant SCZ patient iPSC-derived NSCs. Overexpression of TLX or inhibition of miR-219 could rescue the reduced cell proliferation in *DISC1*-mutant SCZ NSCs.

This study identified an unexpected role for TLX in miRNA maturation at the post-transcriptional level beyond transcriptional regulation. In an unbiased search for TLX-interacting proteins, we identified the RNA helicase p68, a component of the miRNA processing machinery, as a novel TLX-interacting protein. Further study revealed that TLX also interacts with p68-associated Drosha and DGCR8, the two main components of miRNA processing machinery. We show here that TLX inhibits miR-219 processing by interacting with the p68/Drosha/DGCR8 complex, which in turn prevents the miRNA processing machinery from binding to miR-219 primary form. Either knockdown of TLX or blocking the interaction between TLX and the miRNA processing machinery resulted in potent induction of pre-miR-219 and mature miR-219 expression, but had minimal effect on pri-miR-219 expression. The concept that a transcription factor like TLX can participate in post-transcriptional regulation of gene expression may serve as a general paradigm for many of these classes of cellular factors to control cell-fate determination.

We detected robust inhibition of cell proliferation and induction of neuronal differentiation when miR-219 was overexpressed in NSCs. However, no obvious change in cell proliferation and differentiation was observed in NSCs treated with miR-219 inhibitor, presumably because the basal miR-219 expression level is low in NSCs. In TLX siRNA-treated NSCs, where miR-219 expression level was elevated, inhibition of miR-219 was able to rescue the proliferative defect and precocious differentiation. It is also possible that the action of other miRNAs, such as miR-9, miR-124, miR-137, miR-338 or let-7, could compensate for miR-219 inhibition in NSCs. miR-219 has been shown to induce oligodendrocyte differentiation in electroporated mouse brains that were collected at E17.5 (ref. 27). However, we did not detect the induction of oligodendrocyte marker expression in miR-219-electroporated mouse brains collected at E15.5, presumably because we collected brains at an earlier stage that is active for neurogenesis but not for gliogenesis yet. It is possible that miR-219 could play distinct roles at different developmental stages.

miR-219 is dysregulated in neurodevelopmental disorders, including SCZ, bipolar disorder and depression^29^,^32^,^42^,^43^. Understanding the regulation of *miR-219* expression in mammalian brains will not only broaden our knowledge about neurodevelopment, but also provide insights into the pathogenesis of neurological disorders. In this study, we show that TLX represses *miR-219* biogenesis in NSCs during mouse brain development. We also identified *PDGFRα* as a downstream target of the TLX-miR-219 cascade in NSCs. PDGFRα has been shown to be expressed in oligodendrocyte progenitor cells^44^ and play a role in oligodendrocyte differentiation downstream of miR-219 (ref. 26). We show here that knockdown of *PDGFRα* expression induced NSC phenotypes similar to that induced by *miR-219* overexpression, whereas overexpression of *PDGFRα* restored NSC phenotypes induced by miR-219 overexpression or TLX siRNA treatment.

DISC1 is required for mouse NSC proliferation^40^. However, little is known about its function in human NSCs. In this study, we have found a role for DISC1 in regulating human NSC proliferation by studying NSCs derived from *DISC1*-mutant SCZ patient iPSCs and genetically engineered isogenic iPSCs with an introduced *DISC1* mutation. Our observation that *miR-219* expression is upregulated, whereas *TLX* expression is downregulated, in *DISC1*-mutant NSCs provides a direct link between TLX and miR-219 expression and DISC1 function. Previous studies have shown that *TLX* KO mice exhibit neuroanatomical and behavioural abnormalities similar to that in *DISC1*-mutant mice and SCZ patients, including increased lateral ventricles, reduced cerebral cortex, reduced neurogenesis and memory, and increased anxiety and hyperactivity^2^,^3^,^6^,^7^,^45^,^46^,^47^,^48^,^49^,^50^,^51^,^52^,^53^,^54^,^55^,^56^. Our result of altered expression of TLX in *DISC1*-mutant NSCs suggests that mutant DISC1 could regulate TLX expression, which in turn induces abnormal miR-219 expression and inhibition of NSC proliferation.

SCZ is a neurodevelopmental disorder for which the pathological mechanism remains elusive. Increasing evidence suggests that miRNAs may play important roles in the aetiology of SCZ^57^. miRNA-219 is highly upregulated in the prefrontal cortex of SCZ patients^29^,^32^ and mediates the behavioural effects of the NMDA receptor antagonist dizocilpine^58^. However, whether miR-219 plays a role in SCZ pathogenesis remained unknown. Our study has identified a novel role for miR-219 in SCZ NSCs; elevated miR-219 expression reduces SCZ NSC proliferation.

Multiple studies provide evidence that NSC proliferation and neurogenesis are tightly linked to SCZ pathogenesis^39^,^59^,^60^. Recent studies using patient iPSCs have identified phenotypic differences in human iPSC-derived neural progenitor cells^61^ and provided insights into how risk factors for SCZ regulate NSC phenotypes and neurodevelopment^62^. In this study, we have provided a direct link between *DISC1* mutation and altered *TLX* and *miR-219* expression, and a causative link between dysregulated *TLX* and *miR-219* expression and proliferative defects in *DISC1*-mutant SCZ NSCs. Our study provides a molecular mechanism underlying defective NSC proliferation in SCZ. Moreover, our study suggests that both TLX and miR-219 could be potential therapeutic targets for SCZ, and that TLX inducers or miR-219 inhibitors may serve as potential therapeutic tools to maintain normal NSC proliferation in SCZ patients.

## Methods

### Animals

Female ICR or Swiss Webster mice at gestation 13.5 were used for *in utero* electroporation experiments. All mice were produced in the Animal Resource Core of City of Hope. All animal-related work was performed under the IACUC protocol 03038 approved by City of Hope Institutional Animal Care and Use Committee. Mice were maintained in a 12-h light:12-h dark light cycle at four mice per cage.

### Antibodies and immunostaining

We used antibodies to Flag epitope tag M2 (Sigma-Aldrich, F2426 for IP), HA (1:500, Santa Cruz, sc-805), p68 (1:1,000, Abcam, ab10261), Drosha (1:1,000, Cell Signaling, #3364), DGCR8 (1:500, Proteintech Group, Inc., 10996-1-AP), BrdU (1:5,000, Accurate, OBT0030CX), DCX (1:300, Santa Cruz, sc-8806) and Ki67 (1:200, GeneTex, GTX16667). Immunostaining of embryonic mouse brains was performed using antibodies for DCX and Ki67. For Ki67 staining, antigen retrieval was performed by incubating slides in sodium citrate buffer (10 mM sodium citrate, pH 6.0 and 0.1% Triton X) at 80 °C for 10 min before staining.

### Mouse NSC culture

Embryonic mouse NSCs were prepared using an established protocol^63^ as follows. E14.5 mouse brains were dissociated by gentle pipetting. The dissociated cells were seeded on polyornithine- and fibronectin-coated plates and cultured in N2 medium (DMEM F12, 25 μg ml^−1^ insulin, 100 ng ml^−1^ apo-transferrin, 30 nM sodium selenite, 20 nM progesterone and 100 μM putrescine) supplemented with 10 ng ml^−1^ FGF2. Cells were maintained as mycoplasm-free culture as revealed by routine mycoplasm screen using MycoAlert mycoplasma detection kit. For differentiation, NSCs were dissociated into single cells and cultured in N2 medium supplemented with 0.5% fetal bovine serum (FBS) and 10 μM forskolin for 5 days. For BrdU labelling, 10 μM BrdU was added to NSCs and pulsed for 30 min. Cells were then fixed and acid treated, followed by immunostaining with anti-BrdU antibody. Transfection of NSCs with reporter plasmid DNA, miRNA or siRNA was performed using TransFectin (Bio-Rad), following manufacturer's instructions. For actinomycin D treatment, control or TLX siRNA-transduced NSCs were treated with 1 μM actinomycin D for 3 h, followed by collection of cells and RNA isolation.

### Plasmid DNAs

pCK-Flag-Drosha^64^ and pCK-Flag-DGCR8 (ref. 64) were gifts from Dr VN Kim. To prepare the PDGFRα 3′-UTR reporter construct, we subcloned DNA fragments containing mouse PDGFRα 3′-UTR into psiCHECK vector (Promega). The miR-219-5P target site 5′-GACAATCA-3′ in PDGFRα 3′-UTR was mutated into 5′-GATCGTCA-3′ by site-directed mutagenesis. The cDNA of mouse PDGFRα was purchased from ATCC and subcloned into pEF-pUb-RFP vector^4^. To make TLX siRNA or scrambled control RNA-expressing lentiviral vector, DNA fragments containing TLX siRNA or scrambled control siRNA hairpin sequences were subcloned into pHIV-GFP vector^65^. To prepare the Dpi peptide or control peptide-expressing vector, DNA fragment containing the Dpi (amino acid residues 341–359) or control peptide (amino acid residues 201–223) of TLX was fused in-frame to three copies of nuclear localization signals and cloned into the CMX-HA or CSC-GFP vector^3^. To make miR-219-expressing retroviral vector, DNA oligos of miR-219 were annealed and cloned into the UEG vector^66^. To prepare the construct of TuD-miR-219, DNA oligos of TuD-miR-219, 5′- TCGAAGAATTGCGTTCTGATGGACAATCA -3′ and 5′- CTAGTGATTGTCCATCAGAACGCAATTCT -3′ were annealed and cloned into the U6-TuD vector. The DNA fragment containing the U6 promoter and TuD-miR-219 was then subcloned into pHIV-GFP vector or CMVLV lentiviral vector containing a puromycin-resistant gene^65^. To prepare miR-219-Glo vector, 392-bp fragment of pri-miR-219 including the pre-miR-219 hairpin loop was PCR amplified using the following primers: 5′- TTCATAGAGCTCACACCGGCTTGTCCACCTTAC -3′ and 5′- TTCATACTCGAGGAGGATACGGAAAGAGGCGAG -3′. The PCR product was digested with SacI and XhoI sites and cloned into the pmirGLO vector (Promega). To prepare miR-1224-Glo vector, 398-bp fragment of pri-miR-1224 was PCR amplified using the following primers: 5′- GATAGCTAGCAATGGCAACTCCAAGCGTGCT -3′ and 5′- ATGAGGCCGAGGTGGGGCTGAGTCTAGAGATC -3′. The PCR product was digested with NheI and XbaI and cloned into the pmirGLO vector (Promega).

### siRNAs and miRNAs

All synthetic siRNAs, miRNAs and their controls were purchased from Dharmacon. The ON-TARGET plus siRNA for TLX (J-065577-12-0005) and non-targeting control siRNA (D-001810-01-05) were used for the experiments of TLX knockdown; the ON-TARGET plus SMARTpool siRNA for PDGFRα (L-048730-00-0005) and control siRNA pool (D-001810-10-05) were used for the experiments of PDGFRα knockdown. The miRIDIAN miRNA mimic for miR-219-5p (c-310578-05-0005), negative control (CN-001000-01-05) and miR-219-5p hairpin inhibitor (IH-310578-07-0005) were used for overexpressing miR-219 or inhibiting miR-219 action in mouse NSCs.

### Northern blot analysis and RT–PCR

Total RNAs from tissue cultured cells or 6- to 8-week-old WT or TLX KO mouse brains were isolated using TRIzol (Invitrogen) in accordance with manufacturer's instructions. Oligonucleotides complementary to miRNA sequences were end-labelled with γ^32^P-ATP and used as probes for northern blot analysis. The sequences for the probes are listed in Supplementary Table 1. RT–PCR was performed to detect the levels of primary, precursor and mature miR-219, or TLX and PDGFRα mRNAs. Reverse transcription was performed using Tetro cDNA synthesis kit (Bioline), and the expression levels of pri-miRNA and pre-miRNA of miR-219, and TLX and PDGFRα mRNAs were determined using DyNAmo Flash SYBR Green qPCR mix (Thermo Scientific) and StepOnePlus Real-Time PCR system (Applied Biosystems). For detection of the mature miR-219, TaqMan MicroRNA assay kit (Applied Biosystems) was used according to manufacturer's protocol. Data analysis was done by comparative Ct method. Results were normalized to β-actin for pri-miRNA, pre-miRNA, TLX and PDGFRα mRNAs, and snoRNA or U6 for mature miRNA. The primers are listed in Supplementary Table 1.

### *In vivo* monitoring of pri-miRNA processing

The miR-219-Glo, miR-1224-Glo or control-Glo (pmirGLO, Promega) vector was transfected together with TLX-expressing vector or TLX siRNA-expressing vector. The firefly luciferase activity was measured 48 h after transfection and normalized with the *Renilla* luciferase internal control. The results were then normalized with the luciferase activity in cells transfected with the control vector(s).

### Nuclear extract preparation and immunoprecipitation

To make stable HeLa cell line that express HA or HA-TLX, we transduced HeLa cells with lentivirus expressing HA or HA-TLX and a GFP reporter, and plated the transduced cells at one cell per well in a 96-well plate. The GFP-positive clones derived from single GFP-positive cells were expanded to make stable cell lines. The expression of HA-TLX in cells transduced with the HA-TLX-expressing virus was confirmed by TLX western blot analysis. Nuclear extracts were prepared from the stable cell line expressing HA or HA-TLX following a published method^67^. Every millilitre of nuclear extract was pre-cleared using 20 μl of protein G and 20 μl of IgG-AC (Santa Cruz, sc-2345) for 5 h, and then incubated with HA beads (Santa Cruz, sc-805 AC) at 4 °C for overnight. Proteins pulled down by the HA beads were collected at 8,200 g for 1 min, then washed with 500 μl TBS for 20 min twice and then re-suspended in protein-loading buffer for protein gel electrophoresis and subsequent MS analysis.

### Mass spectrometry

Proteins were separated on a 4–12% Bis-Tris NuPAGE gel with MES running buffer (Novex, Life Technologies) and stained with SimplyBlue SafeStain solution (Life Technologies) to visualize the differentially expressed proteins. Corresponding protein gel bands were excised and destained in ammonium bicarbonate (100 mM)/acetonitrile (45%) followed by in-gel processing, which included reduction with Tris(carboxyethyl) phosphine (10 mM), alkylation with iodoacetamide (50 mM) and digestion with sequencing grade trypsin (300 ng per band, Promega); all steps were performed in 100 mM ammonium bicarbonate, pH 7.9. Extracted peptides were acidified with formic acid (1%) and injected straightly into the liquid chromatography (LC) MS system, consisting of a binary pump Agilent 1200 HPLC, a 6,520 quadrupole time-of-flight mass spectrometer (Agilent), equipped with a chip cube ion source, utilizing a high-capacity LC/MS chip (Agilent) with a 150 mm × 75 μm Zorbax 300SB-C18 on-board analytical reverse phase column and a 160-nl trapping column. Approximately, 10 μl sized peptide samples were loaded at 4 μl per min. LC was performed with a gradient mobile phase system containing buffer A (0.1% aqueous formic acid) and B (100% acetonitrile, 0.1% formic acid). A 50-min gradient elution from the analytical column was conducted from 7 to 85% buffer B at 300 nl min^−1^. MS and tandem MS analysis of peptide ions with z >2^+^ was performed in data-dependent mode. Automated collision energy settings were set by the acquisition software, MassHunter (Agilent). The resulting data were analysed using the GPM X! Tandem search engine (The Global Proteome Machine Organization) with the human protein database and Scaffold (Proteome Software) at a 1% false discovery rate setting.

### Immunoprecipitation and western blotting

Cells were lysed with lysis buffer containing 50 mM Tris-HCl (pH 7.8), 150 mM NaCl, 1% NP-40, 0.1% deoxycholate and protease inhibitor cocktails (Roche). For DNase and RNase treatment, cell lysates were treated with 40 U ml^−1^ DNase and 10 μg ml^−1^ RNase at 37 °C for 30 min. Lysates were immunoprecipitated using anti-Flag (Sigma-Aldrich, F2426) or anti-HA (Santa Cruz, sc-805 AC) antibodies. For co-immunoprecipitation of endogenous proteins, E13.5 mouse brains were homogenized in the above lysis buffer. Lysates were immunoprecipitated using TLX antibody, followed by immunoblotting using indicated antibodies. To determine whether Dpi disrupts the TLX-Drosha interaction, constructs expressing Dpi (TLX residues 341–359) or a control peptide (TLX residues 201–223), together with HA-TLX and Flag-Drosha were transfected into HEK293T cells. Cell lysates were immunoprecipitated with Flag antibody (Sigma-Aldrich, F2426), followed by immunoblotting with anti-HA (1:500, Santa Cruz, sc-805) or anti-Flag antibody (1:500, Sigma-Aldrich, F1804). Images in Figs 1a,b, 2c,d,f and 5b–e have been cropped for presentation. Full size images are presented in Supplementary Figs 8–10.

### RNA immunoprecipitation

RNA immunoprecipitation was performed under native condition^68^. NSCs were transduced with lentivirus expressing TLX siRNA or scrambled control RNA. Cell pellets were re-suspended in ice-cold lysis buffer containing 100 mM KCl, 5 mM MgCl_2_, 10 mM HEPES (pH 7.0), 0.5% NP-40, 1 mM dithiothreitol (DTT), 100 U per ml RNase inhibitor (Promega) and protease inhibitor cocktail (Roche), and lysates were passed through a 27.5-gauge needle four times to promote nuclear lysis. Eighty units of DNase (Ambion) was added to the lysates, which were then incubated on ice for 30 min. Cell lysates were diluted in NT2 buffer (50 mM Tris-HCl (pH 7.4), 150 mM NaCl, 1 mM MgCl_2_, 0.05% NP-40, 1 mM DTT, RNase inhibitor and protease inhibitor cocktail) and pre-cleared with protein G agarose. One-tenth volume of the supernatant was saved as input. The rest of the supernatant was incubated with 5 μg of antibodies at 4 °C for overnight. After incubating with protein G agarose, the RNA-antibody complex was precipitated and washed with NT2 buffer four times. RNA was extracted using TRIzol (Invitrogen) according to manufacturer's instructions. Reverse transcription was performed using Tetro cDNA synthesis kit (Bioline), followed by PCR.

### *In utero* electroporation

A solution including 100 μM miRNA, siRNA or miRNA inhibitor with 5 μg μl^−1^ plasmid DNA expressing RFP only or RFP and PDGFRα was electroporated into E13.5 WT ICR mouse brains. Both male and female mice were used. For electroporation of the Dpi peptide-expressing vector, plasmids expressing Dpi and RFP or Dpi, RFP and TuD-miR-219, at 2.5 μg μl^−1^ each, were electroporated into E13.5 mouse brains. Two days later, the electroporated brains were dissected and sectioned at 20-μm thickness, followed by immunostaining.

### Human iPSC culture and differentiation

Human iPSCs were maintained and cultured in Essential 8 (E8) medium (Gibco, A15169-01). For NSC differentiation, iPSCs were detached using 0.5 mM EDTA and cultured in E8 medium for 6 days in suspension for embryoid body (EB) formation, then switched to neuronal induction medium (50% DMEM/F12, 50% Neurobasal, 0.5 × N2, 0.5 × B27, 2 mM L-glutamine, 0.1 mM non-essential amino acids (NEAA) and 100 units penicillin/streptomycin) supplemented with 5 μM SB431542 and 0.25 μM LDN for 3 days. The EB spheres were transferred into Matrigel-coated plates and cultured in neuronal induction medium for 7 days. Rosette structures were mechanically lifted and cultured in neuronal induction medium supplemented with basic FGF (5 ng per ml) and EGF (20 ng ml^−1^) for expansion. Neurospheres were stained for NSC markers using antibodies for SOX1 (1:500, Millipore, AB15766) and NESTIN (1:1,000, BD, 611659). All the cells used in this study were maintained as mycoplasm-free culture as revealed by routine mycoplasm screen using MycoAlert mycoplasma detection kit.

### Human NSC proliferation and differentiation

Human iPSC-derived NSCs were seeded on Matrigel-coated 24-well plates in proliferation media and cultured for 24 h. Lentivirus expressing miR-219 or TuD-miR-219 and a GFP reporter was added to human NSCs in 24-well plates for 16 h. The virus-transduced cells were labelled with GFP. For proliferation assay, cells were allowed to recover for 2 days and then treated with 10 μM BrdU for 1 h, followed by immunostaining for BrdU and SOX1. Nuclei were counter-stained using 4,6-diamidino-2-phenylindole (DAPI). NSC proliferation rate was determined using the percentage of BrdU+SOX1+ cells, which was calculated as BrdU+SOX1+/DAPI+ cells for non-virus-transduced cells and BrdU+SOX1+GFP+/GFP+ cells for GFP-expressing virus-transduced cells. For differentiation, NSCs were switched to differentiation medium containing N2 and B27 (1:1) with 1 μM retinoic acid and 0.5% FBS in DMEM F12 media. Cells were allowed to differentiate for 2 weeks, followed by immunostaining for Tuj1. The neuronal differentiation rate was determined using the percentage of Tuj1+ cells, which was calculated as Tuj1+/DAPI+ cells for non-virus-transduced cells and Tuj1+GFP+/GFP+ cells for GFP-expressing virus-transduced cells.

### Statistical analysis

Student's *t*-test and analysis of variance (ANOVA) were used for statistical analyses for comparison of experimental results as reported in each figure and legend. All results were expressed as mean±s.d. The sample size was chosen based on our preliminary studies. Statistical significance was defined as *P*<0.05, *P*<0.01 or *P*<0.001 as specified in the figure legend. No samples, mice or data points were excluded from the reported analyses. No randomization was used for sample assignment and data collection, and no blinding was performed.

## Additional information

**How to cite this article:** Murai, K. *et al*. The TLX-miR-219 cascade regulates neural stem cell proliferation in neurodevelopment and schizophrenia iPSC model. *Nat. Commun.* 7:10965 doi: 10.1038/ncomms10965 (2016).

## Supplementary Material

Supplementary Information Supplementary Figures 1-10 and Supplementary Table 1

## References

[b1] Yu R. T., McKeown M., Evans R. M. & Umesono K. Relationship between Drosophila gap gene tailless and a vertebrate nuclear receptor Tlx. Nature 370, 375–379 (1994) . 8047143 10.1038/370375a0

[b2] Monaghan A. P. . Defective limbic system in mice lacking the tailless gene. Nature 390, 515–517 (1997) . 9394001 10.1038/37364

[b3] Shi Y. . Expression and function of orphan nuclear receptor TLX in adult neural stem cells. Nature 427, 78–83 (2004) . 14702088 10.1038/nature02211

[b4] Li W. . Nuclear receptor TLX regulates cell cycle progression in neural stem cells of the developing brain. Mol. Endocrinol. 22, 56–64 (2008) . 17901127 10.1210/me.2007-0290 PMC2194628

[b5] Qu Q. . Orphan nuclear receptor TLX activates Wnt/beta-catenin signalling to stimulate neural stem cell proliferation and self-renewal. Nat. Cell Biol. 12, 31–40; sup pp 31–39 (2010) . 20010817 10.1038/ncb2001 PMC2880892

[b6] Zhang C. L., Zou Y., He W., Gage F. H. & Evans R. M. A role for adult TLX-positive neural stem cells in learning and behaviour. Nature 451, 1004–1007 (2008) . 18235445 10.1038/nature06562

[b7] Murai K. . Nuclear receptor TLX stimulates hippocampal neurogenesis and enhances learning and memory in a transgenic mouse model. Proc. Natl Acad. Sci. USA 111, 9115–9120 (2014) . 24927526 10.1073/pnas.1406779111 PMC4078800

[b8] Roy K. . The Tlx gene regulates the timing of neurogenesis in the cortex. J. Neurosci. 24, 8333–8345 (2004) . 15385616 10.1523/JNEUROSCI.1148-04.2004 PMC2740800

[b9] Stenman J. M., Wang B. & Campbell K. Tlx controls proliferation and patterning of lateral telencephalic progenitor domains. J. Neurosci. 23, 10568–10576 (2003) . 14627641 10.1523/JNEUROSCI.23-33-10568.2003 PMC6740920

[b10] Zhao C. . microRNA let-7b regulates neural stem cell proliferation and differentiation by targeting nuclear receptor TLX signaling. Proc. Natl Acad. Sci. USA 107, 1876–1881 (2010) . 20133835 10.1073/pnas.0908750107 PMC2836616

[b11] Sun G., Yu R. T., Evans R. M. & Shi Y. Orphan nuclear receptor TLX recruits histone deacetylases to repress transcription and regulate neural stem cell proliferation. Proc. Natl Acad. Sci. USA 104, 15282–15287 (2007) . 17873065 10.1073/pnas.0704089104 PMC2000559

[b12] Sun G. . Histone demethylase LSD1 regulates neural stem cell proliferation. Mol. Cell. Biol. 30, 1997–2005 (2010) . 20123967 10.1128/MCB.01116-09 PMC2849468

[b13] Yokoyama A., Takezawa S., Schule R., Kitagawa H. & Kato S. Transrepressive function of TLX requires the histone demethylase LSD1. Mol. Cell. Biol. 28, 3995–4003 (2008) . 18391013 10.1128/MCB.02030-07 PMC2423124

[b14] Zhao C., Sun G., Li S. & Shi Y. A feedback regulatory loop involving microRNA-9 and nuclear receptor TLX in neural stem cell fate determination. Nat. Struct. Mol. Biol. 16, 365–371 (2009) . 19330006 10.1038/nsmb.1576 PMC2667220

[b15] Iwahara N., Hisahara S., Hayashi T. & Horio Y. Transcriptional activation of NAD+-dependent protein deacetylase SIRT1 by nuclear receptor TLX. Biochem. Biophys. Res. Commun. 386, 671–675 (2009) . 19555662 10.1016/j.bbrc.2009.06.103

[b16] Elmi M. . TLX activates MASH1 for induction of neuronal lineage commitment of adult hippocampal neuroprogenitors. Mol. Cell. Neurosci. 45, 121–131 (2010) . 20599619 10.1016/j.mcn.2010.06.003

[b17] Bartel D. P. MicroRNAs: genomics, biogenesis, mechanism, and function. Cell 116, 281–297 (2004) . 14744438 10.1016/s0092-8674(04)00045-5

[b18] Fukuda T. . DEAD-box RNA helicase subunits of the Drosha complex are required for processing of rRNA and a subset of microRNAs. Nat. Cell Biol. 9, 604–611 (2007) . 17435748 10.1038/ncb1577

[b19] Gregory R. I. . The Microprocessor complex mediates the genesis of microRNAs. Nature 432, 235–240 (2004) . 15531877 10.1038/nature03120

[b20] Fuller-Pace F. V. & Ali S. The DEAD box RNA helicases p68 (Ddx5) and p72 (Ddx17): novel transcriptional co-regulators. Biochem. Soc. Trans. 36, 609–612 (2008) . 18631126 10.1042/BST0360609

[b21] Davis B. N., Hilyard A. C., Lagna G. & Hata A. SMAD proteins control DROSHA-mediated microRNA maturation. Nature 454, 56–61 (2008) . 18548003 10.1038/nature07086 PMC2653422

[b22] Suzuki H. I. . Modulation of microRNA processing by p53. Nature 460, 529–533 (2009) . 19626115 10.1038/nature08199

[b23] Kawai S. & Amano A. BRCA1 regulates microRNA biogenesis via the DROSHA microprocessor complex. J. Cell Biol. 197, 201–208 (2012) . 22492723 10.1083/jcb.201110008 PMC3328391

[b24] Cheng H. Y. . microRNA modulation of circadian-clock period and entrainment. Neuron 54, 813–829 (2007) . 17553428 10.1016/j.neuron.2007.05.017 PMC2590749

[b25] Lukiw W. J. Micro-RNA speciation in fetal, adult and Alzheimer's disease hippocampus. Neuroreport 18, 297–300 (2007) . 17314675 10.1097/WNR.0b013e3280148e8b

[b26] Dugas J. C. . Dicer1 and miR-219 are required for normal oligodendrocyte differentiation and myelination. Neuron 65, 597–611 (2010) . 20223197 10.1016/j.neuron.2010.01.027 PMC2843397

[b27] Zhao X. . MicroRNA-mediated control of oligodendrocyte differentiation. Neuron 65, 612–626 (2010) . 20223198 10.1016/j.neuron.2010.02.018 PMC2855245

[b28] Hudish L. I., Blasky A. J. & Appel B. miR-219 regulates neural precursor differentiation by direct inhibition of apical par polarity proteins. Dev. Cell. 27, 387–398 (2013) . 24239515 10.1016/j.devcel.2013.10.015 PMC3862977

[b29] Beveridge N. J., Gardiner E., Carroll A. P., Tooney P. A. & Cairns M. J. Schizophrenia is associated with an increase in cortical microRNA biogenesis. Mol. Psychiatry 15, 1176–1189 (2010) . 19721432 10.1038/mp.2009.84 PMC2990188

[b30] Beveridge N. J. . Dysregulation of miRNA 181b in the temporal cortex in schizophrenia. Hum. Mol. Genet. 17, 1156–1168 (2008) . 18184693 10.1093/hmg/ddn005

[b31] Santarelli D. M., Beveridge N. J., Tooney P. A. & Cairns M. J. Upregulation of dicer and microRNA expression in the dorsolateral prefrontal cortex Brodmann area 46 in schizophrenia. Biol. Psychiatry 69, 180–187 (2011) . 21111402 10.1016/j.biopsych.2010.09.030

[b32] Smalheiser N. R. . Expression of microRNAs and other small RNAs in prefrontal cortex in schizophrenia, bipolar disorder and depressed subjects. PLoS ONE 9, e86469 (2014) . 24475125 10.1371/journal.pone.0086469 PMC3903529

[b33] Berezikov E., Chung W. J., Willis J., Cuppen E. & Lai E. C. Mammalian mirtron genes. Mol. Cell 28, 328–336 (2007) . 17964270 10.1016/j.molcel.2007.09.028 PMC2763384

[b34] Jackson E. L. . PDGFR alpha-positive B cells are neural stem cells in the adult SVZ that form glioma-like growths in response to increased PDGF signaling. Neuron 51, 187–199 (2006) . 16846854 10.1016/j.neuron.2006.06.012

[b35] Haraguchi T., Ozaki Y. & Iba H. Vectors expressing efficient RNA decoys achieve the long-term suppression of specific microRNA activity in mammalian cells. Nucleic Acids Res. 37, e43 (2009) . 19223327 10.1093/nar/gkp040 PMC2665227

[b36] Sachs N. A. . A frameshift mutation in disrupted in schizophrenia 1 in an American family with schizophrenia and schizoaffective disorder. Mol. Psychiatry 10, 758–764 (2005) . 15940305 10.1038/sj.mp.4001667

[b37] Chiang C. H. . Integration-free induced pluripotent stem cells derived from schizophrenia patients with a DISC1 mutation. Mol. Psychiatry 16, 358–360 (2011) . 21339753 10.1038/mp.2011.13 PMC4005725

[b38] Wen Z. . Synaptic dysregulation in a human iPS cell model of mental disorders. Nature 515, 414–418 (2014) . 25132547 10.1038/nature13716 PMC4501856

[b39] Reif A. . Neural stem cell proliferation is decreased in schizophrenia, but not in depression. Mol. Psychiatry 11, 514–522 (2006) . 16415915 10.1038/sj.mp.4001791

[b40] Mao Y. . Disrupted in schizophrenia 1 regulates neuronal progenitor proliferation via modulation of GSK3beta/beta-catenin signaling. Cell 136, 1017–1031 (2009) . 19303846 10.1016/j.cell.2008.12.044 PMC2704382

[b41] Ishizuka K. . DISC1-dependent switch from progenitor proliferation to migration in the developing cortex. Nature 473, 92–96 (2011) . 21471969 10.1038/nature09859 PMC3088774

[b42] Sarachana T., Zhou R., Chen G., Manji H. K. & Hu V. W. Investigation of post-transcriptional gene regulatory networks associated with autism spectrum disorders by microRNA expression profiling of lymphoblastoid cell lines. Genome Med. 2, 23 (2010) . 20374639 10.1186/gm144 PMC2873801

[b43] Saus E. . Genetic variants and abnormal processing of pre-miR-182, a circadian clock modulator, in major depression patients with late insomnia. Hum. Mol. Genet. 19, 4017–4025 (2010) . 20656788 10.1093/hmg/ddq316

[b44] Rivers L. E. . PDGFRA/NG2 glia generate myelinating oligodendrocytes and piriform projection neurons in adult mice. Nat. Neurosci. 11, 1392–1401 (2008) . 18849983 10.1038/nn.2220 PMC3842596

[b45] Goldberg J. F. & Chengappa K. N. Identifying and treating cognitive impairment in bipolar disorder. Bipolar Disord. 11, (Suppl 2), 123–137 (2009) . 19538691 10.1111/j.1399-5618.2009.00716.x

[b46] Swayze V. W. 2nd, Andreasen N. C., Alliger R. J., Ehrhardt J. C. & Yuh W. T. Structural brain abnormalities in bipolar affective disorder. Ventricular enlargement and focal signal hyperintensities. Arch. Gen. Psychiatry 47, 1054–1059 (1990) . 2241506 10.1001/archpsyc.1990.01810230070011

[b47] Young K. A. . Fierce: a new mouse deletion of Nr2e1; violent behaviour and ocular abnormalities are background-dependent. Behav. Brain Res. 132, 145–158 (2002) . 11997145 10.1016/s0166-4328(01)00413-2 PMC2862907

[b48] Clapcote S. J. . Behavioral phenotypes of Disc1 missense mutations in mice. Neuron 54, 387–402 (2007) . 17481393 10.1016/j.neuron.2007.04.015

[b49] Hikida T. . Dominant-negative DISC1 transgenic mice display schizophrenia-associated phenotypes detected by measures translatable to humans. Proc. Natl Acad. Sci. USA 104, 14501–14506 (2007) . 17675407 10.1073/pnas.0704774104 PMC1964873

[b50] Koike H., Arguello P. A., Kvajo M., Karayiorgou M. & Gogos J. A. Disc1 is mutated in the 129S6/SvEv strain and modulates working memory in mice. Proc. Natl Acad. Sci. USA 103, 3693–3697 (2006) . 16484369 10.1073/pnas.0511189103 PMC1450143

[b51] Kvajo M. . A mutation in mouse Disc1 that models a schizophrenia risk allele leads to specific alterations in neuronal architecture and cognition. Proc. Natl Acad. Sci. USA 105, 7076–7081 (2008) . 18458327 10.1073/pnas.0802615105 PMC2383956

[b52] Li W. . Specific developmental disruption of disrupted-in-schizophrenia-1 function results in schizophrenia-related phenotypes in mice. Proc. Natl Acad. Sci. USA 104, 18280–18285 (2007) . 17984054 10.1073/pnas.0706900104 PMC2084334

[b53] Niwa M. . Knockdown of DISC1 by *in utero* gene transfer disturbs postnatal dopaminergic maturation in the frontal cortex and leads to adult behavioral deficits. Neuron 65, 480–489 (2010) . 20188653 10.1016/j.neuron.2010.01.019 PMC3084528

[b54] Pletnikov M. V. . Enlargement of the lateral ventricles in mutant DISC1 transgenic mice. Mol. Psychiatry 13, 115 (2008) . 18202691 10.1038/sj.mp.4002144

[b55] Shen S. . Schizophrenia-related neural and behavioral phenotypes in transgenic mice expressing truncated Disc1. J. Neurosci. 28, 10893–10904 (2008) . 18945897 10.1523/JNEUROSCI.3299-08.2008 PMC6671369

[b56] Ellison-Wright I., Glahn D. C., Laird A. R., Thelen S. M. & Bullmore E. The anatomy of first-episode and chronic schizophrenia: an anatomical likelihood estimation meta-analysis. Am. J. Psychiatry 165, 1015–1023 (2008) . 18381902 10.1176/appi.ajp.2008.07101562 PMC2873788

[b57] Olde Loohuis N. F. . MicroRNA networks direct neuronal development and plasticity. Cell. Mol. Life Sci. 69, 89–102 (2012) . 21833581 10.1007/s00018-011-0788-1 PMC3249201

[b58] Kocerha J. . MicroRNA-219 modulates NMDA receptor-mediated neurobehavioral dysfunction. Proc. Natl Acad. Sci. USA 106, 3507–3512 (2009) . 19196972 10.1073/pnas.0805854106 PMC2651305

[b59] Flagstad P. . Disruption of neurogenesis on gestational day 17 in the rat causes behavioral changes relevant to positive and negative schizophrenia symptoms and alters amphetamine-induced dopamine release in nucleus accumbens. Neuropsychopharmacology 29, 2052–2064 (2004) . 15199377 10.1038/sj.npp.1300516

[b60] Newton S. S. & Duman R. S. Neurogenic actions of atypical antipsychotic drugs and therapeutic implications. CNS Drugs 21, 715–725 (2007) . 17696572 10.2165/00023210-200721090-00002

[b61] Brennand K. . Phenotypic differences in hiPSC NPCs derived from patients with schizophrenia. Mol. Psychiatry 20, 361–368 (2014) . 24686136 10.1038/mp.2014.22 PMC4182344

[b62] Yoon K. J. . Modeling a genetic risk for schizophrenia in iPSCs and mice reveals neural stem cell deficits associated with adherens junctions and polarity. Cell Stem Cell 15, 79–91 (2014) . 24996170 10.1016/j.stem.2014.05.003 PMC4237009

[b63] Sun G. . miR-137 forms a regulatory loop with nuclear receptor TLX and LSD1 in neural stem cells. Nat. Commun. 2, 529 (2011) . 22068596 10.1038/ncomms1532 PMC3298567

[b64] Lee Y. . The nuclear RNase III Drosha initiates microRNA processing. Nature 425, 415–419 (2003) . 14508493 10.1038/nature01957

[b65] Sun G. . Molecular properties, functional mechanisms, and applications of sliced siRNA. Mol. Ther. Nucleic Acids 4, e221 (2015) . 25602583 10.1038/mtna.2014.73 PMC4345305

[b66] Kim J. Y. . DISC1 regulates new neuron development in the adult brain via modulation of AKT-mTOR signaling through KIAA1212. Neuron 63, 761–773 (2009) . 19778506 10.1016/j.neuron.2009.08.008 PMC3075620

[b67] Abmayr S. M., Yao T., Parmely T. & Workman J. L. Preparation of nuclear and cytoplasmic extracts from mammalian cells. Curr. Protoc. Mol. Biol. Chapter 12, Unit 12.1 (2006) . 18265374 10.1002/0471142727.mb1201s75

[b68] Keene J. D., Komisarow J. M. & Friedersdorf M. B. RIP-Chip: the isolation and identification of mRNAs, microRNAs and protein components of ribonucleoprotein complexes from cell extracts. Nat. Protoc. 1, 302–307 (2006) . 17406249 10.1038/nprot.2006.47

