# Supplementary material for: The TLX-miR-219 cascade regulates neural stem cell proliferation in neurodevelopment and schizophrenia iPSC model
Source: Nat Commun. 2016 Mar 11;7:10965. doi: 10.1038/ncomms10965 (PMC4793043; doi:10.1038/ncomms10965)
Supplement: Supplementary Information — Supplementary Figures 1-10 and Supplementary Table 1 [file ncomms10965-s1.pdf]

Supplementary Figures

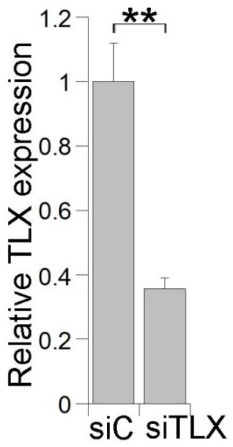

Supplementary Fig. 1 Knockdown of TLX expression in NSCs. Mouse NSCs were transduced with lentivirus expressing a scrambled control RNA (siC) or TLX siRNA (siTLX). The expression of TLX was determined by RT-PCR. Error bars are sd of the mean for all the quantification in this study. n=5. **p< 0.01 by student's t-test.

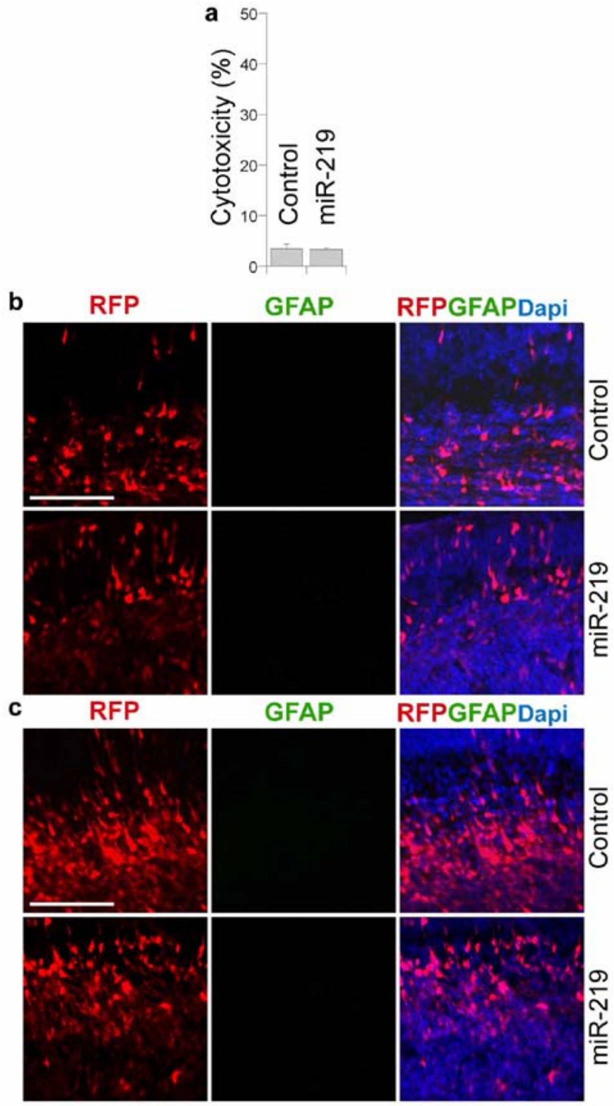

Supplementary Fig. 2 Lack of toxicity and gliogenic induction in miR-219-transfected cells. **a.** Minimal cytotoxicity in miR-219-transfected NSCs. Cytotoxicity was expressed as the percent of lactate dehydrogenase (LDH) release into the medium out of the total LDH activity. n=5. **b, c.** No induction of GFAP and MBP expression in miR-219-electroporated mouse brains at E15.5. Scale bar: 100 µm.

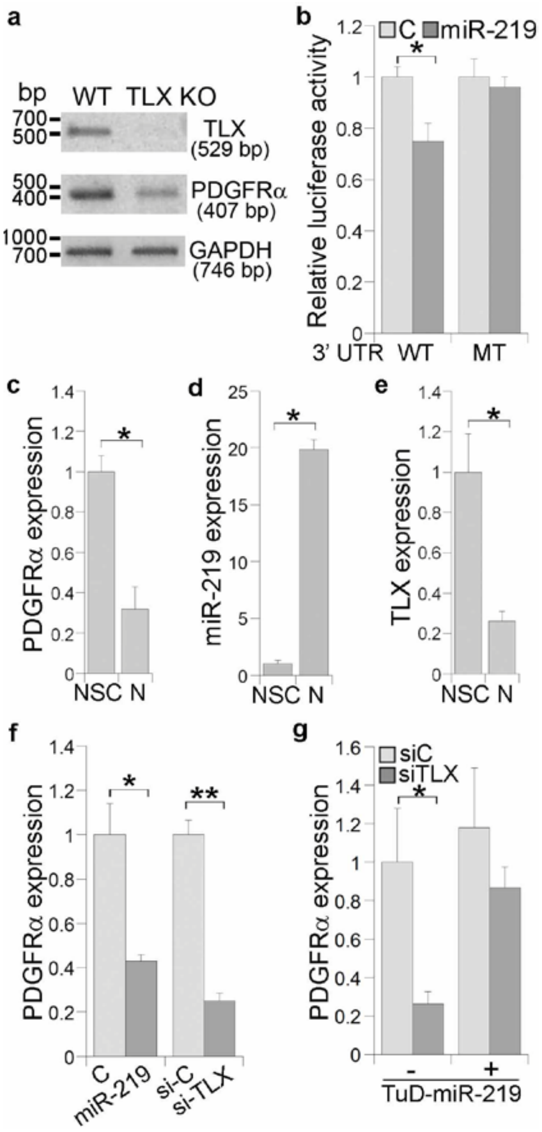

Supplementary Fig. 3 TLX-miR-219 regulates the expression of PDGFRα in NSCs.
a. PDGFRα expression was reduced in TLX KO mouse brains. The expression of PDGFRα in WT and TLX KO mouse brains was examined by RT-PCR. GAPDH was included as a loading control. Size markers are indicated. **b.** miR-219 represses PDGFRα 3′ UTR reporter with wild type (WT), but not mutant (MT) miR-219 recognition sites. n=3. *p< 0.01 by student's t test. **c-e.** The expression pattern of miR-219 in NSCs and neurons inversely correlates with that of PDGFRα and TLX. The expression levels of PDGFRα (c), miR-219 (d) and TLX (e) in NSCs and cortical neurons (N) derived from embryonic mouse brains were determined by RT-PCR. n=3 and *p< 0.001 by student's t test for panels c-e. **f.** The expression of PDGFRα is decreased in NSCs transfected with miR-219 or TLX siRNA as shown by RT-PCR analysis. n=5. *p<0.01, **p<0.001 by student's t test. **g.** Inhibition of PDGFRα expression by TLX siRNA could be rescued by the miR-219 decoy inhibitor, TuD-miR-219. The expression of PDGFRα in NSCs transduced with scramble control RNA (siC) or TLX siRNA (si-TLX), in the absence or presence of TuD-miR-219, was examined by RT-PCR. n=5. *p<0.01 by student's t test.

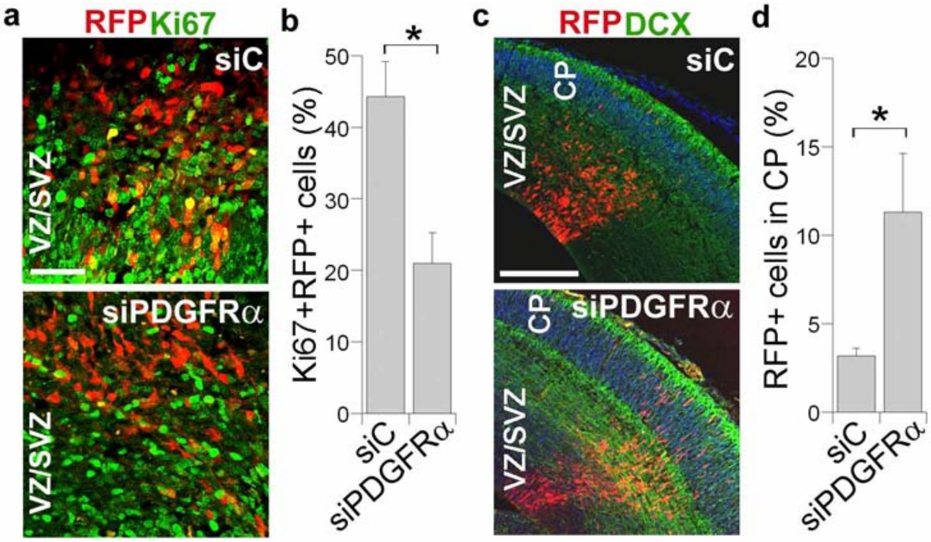

Supplementary Fig. 4 Knockdown of PDGFRα inhibits NSC proliferation *in vivo*.

a. Electroporation of PDGFRα siRNA decreased NSC proliferation in the VZ/SVZ of embryonic mouse brains. The electroporated cells were labeled with RFP and proliferating cells were labeled with Ki67. **b**. The percentage of RFP+Ki67+ cells out of total RFP+ cells in control RNA (siC) or PDGFRα siRNA (siPDGFRα)-electroporated brains is shown. n=3. *$p < 0.05$ by student's t-test. **c**. Electroporation of PDGFRα siRNA led to precocious outward cell migration. The electroporated brains were labeled by RFP and stained for the neuronal marker doublecortin (DCX). **d**. The percentage of electroporated cells (RFP+) that migrated to the CP in siC and siPDGFRα-electroporated brains. n=3. *$p < 0.05$ by student's t test. Scale bar: 50 μm for panel a; 200 μm for panel c.

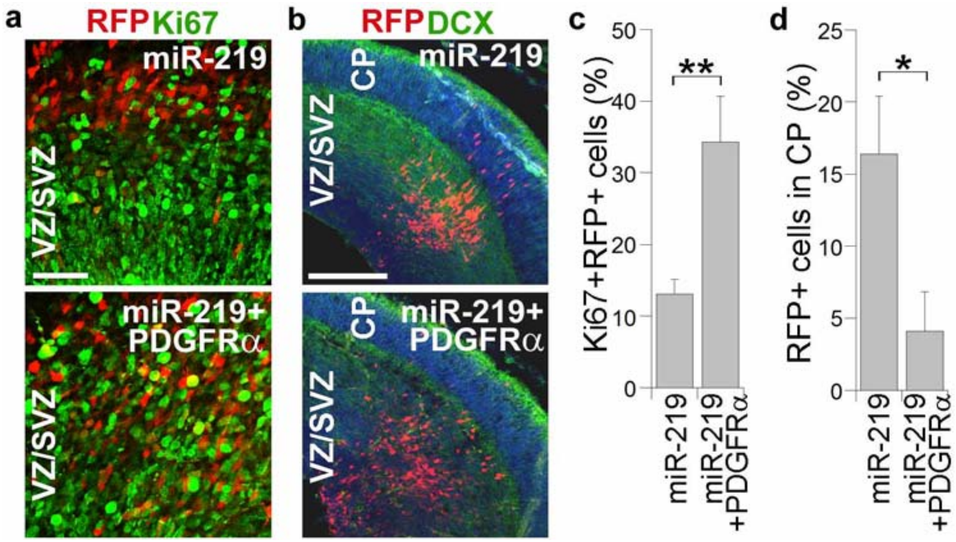

Supplementary Fig. 5 PDGFRα functions downstream of miR-219 in mouse brains. a. Co-electroporation with PDGFRα and miR-219 reversed the decrease in NSC proliferation induced by miR-219 in the VZ/SVZ of embryonic mouse brains. The electroporated cells were labeled with RFP and proliferating cells were labeled with Ki67. **b**. Co-electroporation with PDGFRα and miR-219 reversed precocious outward cell migration induced by electroporation with miR-219 alone. **c**, **d**. The percentage of RFP+Ki67+ cells (c) or cells migrated to the CP (d) out of total RFP+ cells in miR-219 or miR-219 and PDGFRα-electroporated brains is shown. n=3 for panels c & d. *$p < 0.05$, **$p < 0.01$ by student's t-test. Scale bar: 50 µm for panel a; 200 µm for panel b.

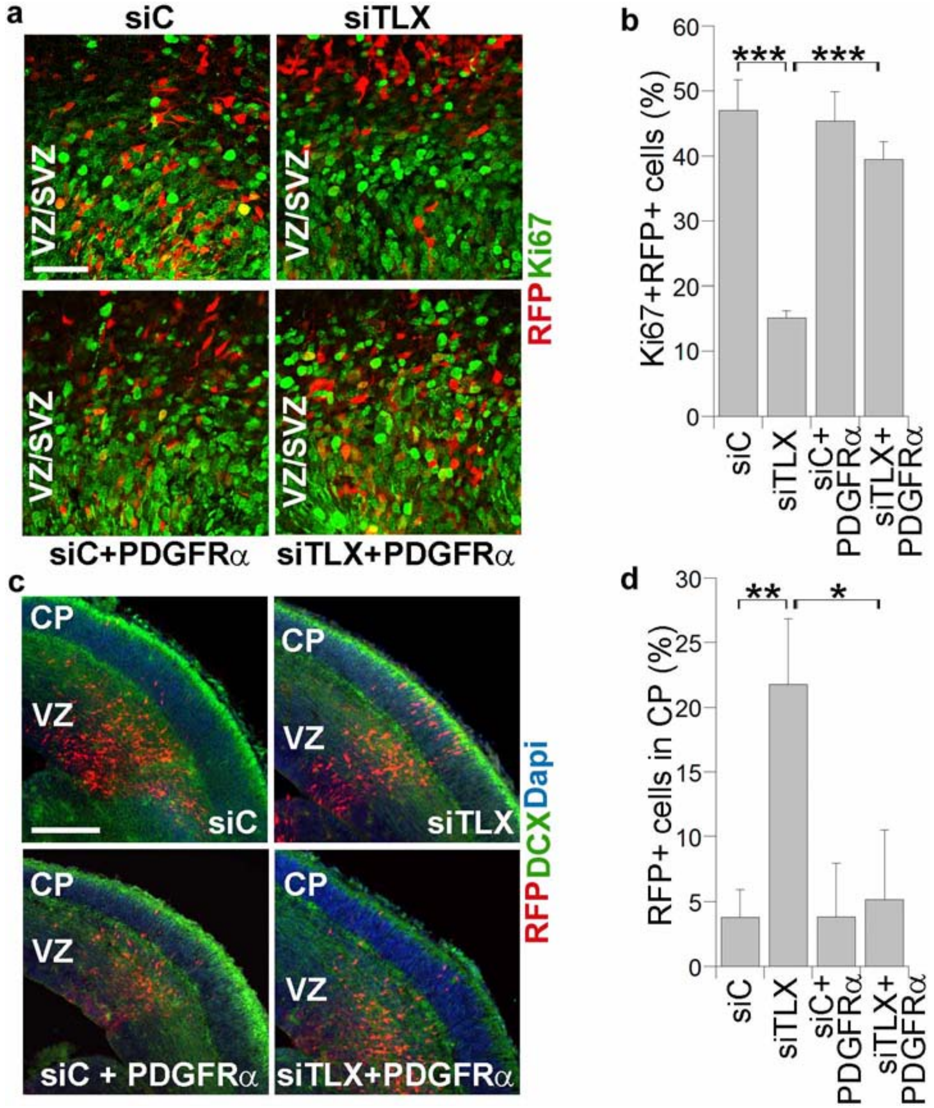

Supplementary Fig. 6 PDGFRα functions downstream of TLX in mouse brains. a. Co-electroporation with PDGFRα and TLX siRNA (siTLX + PDGFRα) reversed the decrease in NSC proliferation in the VZ/SVZ induced by TLX siRNA alone. A control RNA (siC) was included as a negative control for TLX siRNA. **b**. The percentage of RFP+Ki67+ cells out of total RFP+ cells is shown. n=3. **c**. Co-electroporation with PDGFRα and TLX siRNA reversed the outward cell migration induced by TLX siRNA alone. The electroporated cells were labeled by RFP. **d**. The percentage of RFP+ cells that migrated to the CP out of total RFP+ cells is shown. n=3. *$p< 0.05$, **$p< 0.01$, ***$p< 0.001$ by student's t test. Scale bar: 50 µm for panel a; 200 µm for panel b.

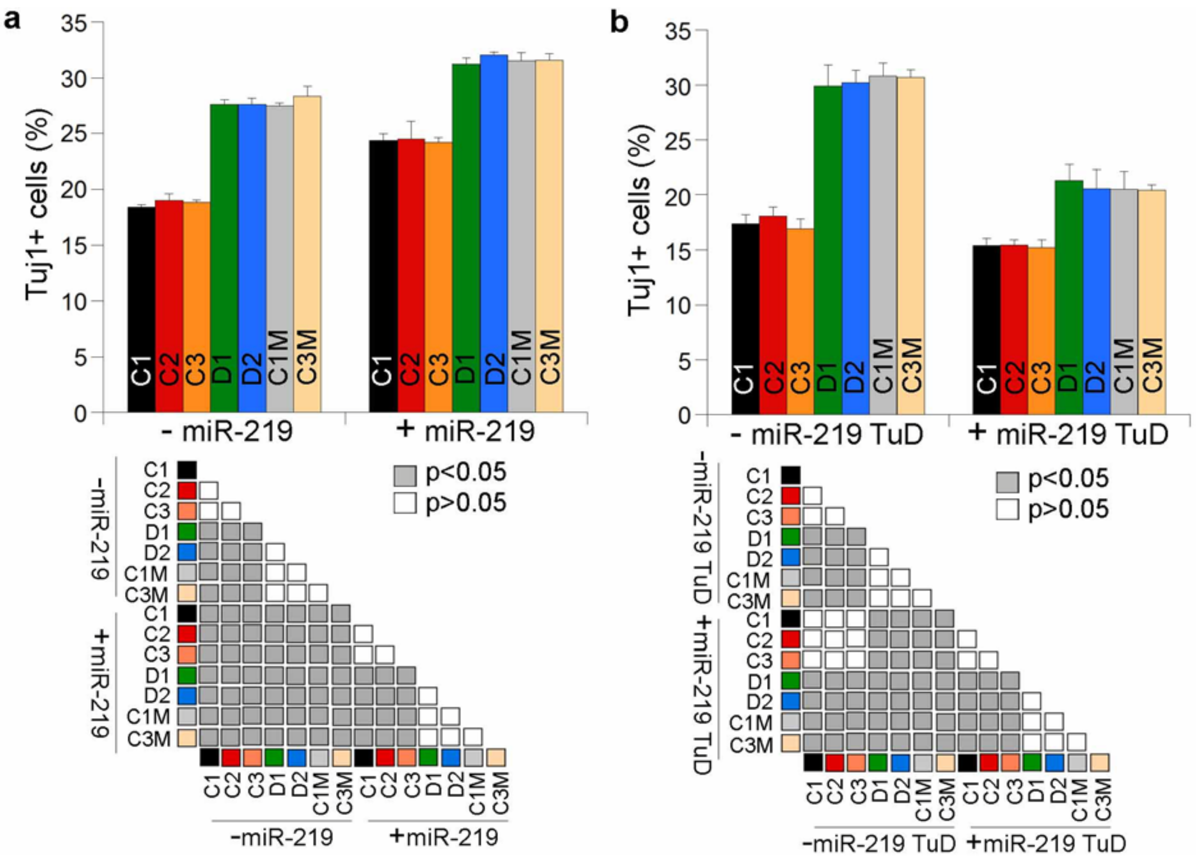

Supplementary Fig. 7 miR-219 regulates neuronal differentiation in SCZ NSCs.
a. Overexpression of miR-219 promotes neuronal differentiation in WT NSCs. WT (C1, C2, C3) and *DISC1*-mutant NSCs (D1, D2, C1M, C3M) were transduced with virus expressing a control vector (-miR-219) or miR-219-expresing vector (+miR-219). Neuronal differentiation rate was determined by the percentage of Tuj1+ cells. **b.** Inhibition of miR-219 reverses precocious neuronal differentiation in SCZ NSCs. The *DISC1*-mutant NSCs exhibited precocious differentiation, which was reversed by TuD-miR-219. Neuronal differentiation rate was determined by the percentage of Tuj1+ cells. n=5 for panels a & b. N represents experimental repeats. ANOVA test result was shown below the graph.

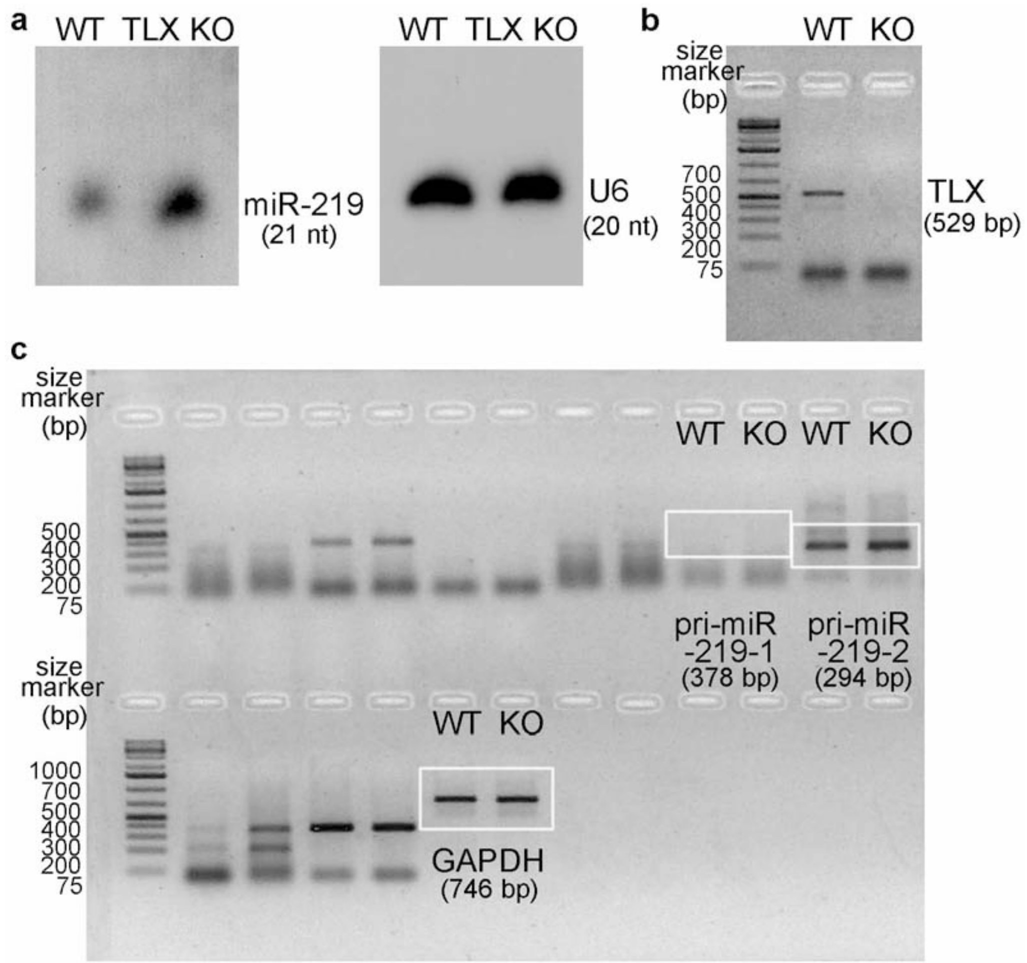

Supplementary Fig. 8 Full gels for images in Fig. 1a &b. a. Full gels for Fig. 1a. The size of the RNA is labeled. **b, c.** Full gels for Fig. 1b. Size markers are included. KO stands for TLX KO.

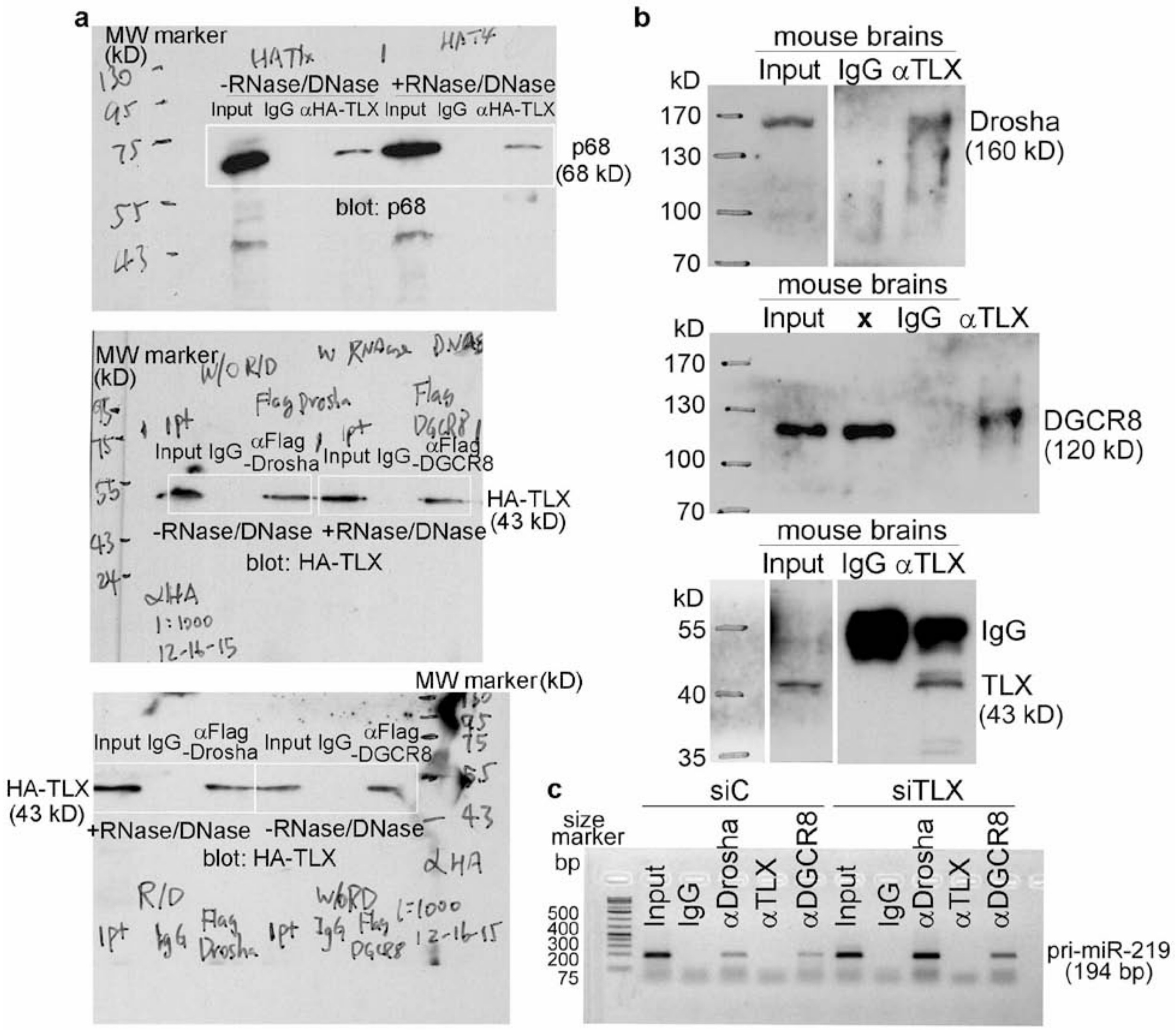

Supplementary Fig. 9 Full gels for images in Fig. 2c, d & f. a. Full gels for Fig. 2c. **b.** Full gels for Fig. 2d. Molecular weight markers are included for panels a and b. **c.** Full gels for Fig. 2f. Size markers are included.

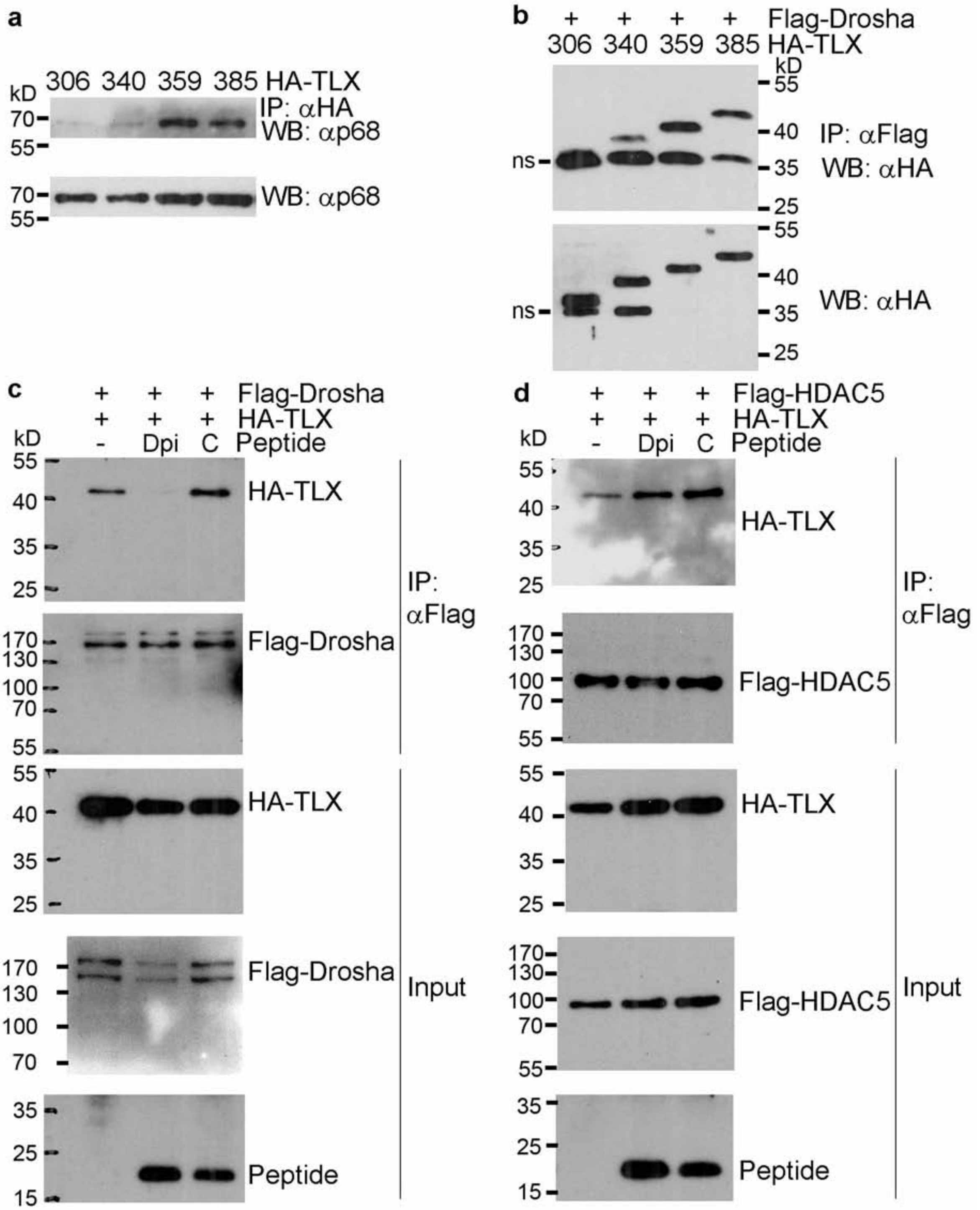

Supplementary Fig. 10 Full gels for images in Fig. 5b-e. a. Gels for Fig. 5b. Molecular weight markers are indicated. **b.** Full gels for Fig. 5c. Molecular weight markers are included. **c, d.** Full gels for Fig. 5d, e. Molecular weight markers are included.

Supplementary Table 1. The list of Northern blot probes and RT-PCR primers

Gene	Strand	Sequence	Assay
miR-219	Antisense	5'-AGAATTGCGTTTGGACAATCA-3'	Northern blot
U6	Antisense	5'-TATGGAACGCTTCTCGAATT-3'	Northern blot
mTLX	Forward	5'-GTCTTTACAAGATCAGCTGATG-3'	RT-PCR
	Reverse	5'-ATGTCACTGGATTTGTACATATC-3'	RT-PCR
pri-miR-219-1	Forward	5'-TTTCCCACGCCAGACATTCAC-3'	RT-PCR
	Reverse	5'-GATCCCCAACTTCTCTCAAGC-3'	RT-PCR
pri-miR-219-2	Forward	5'- TTGCCGAGCTTCTGCGAGGTA-3'	RT-PCR
	Reverse	5'- TGTCCCCTCTTTGCATGCCAG-3'	RT-PCR
PDGFRα	Forward	5'-CAAACCTGACCATGCCACCAG-3'	RT-PCR
	Reverse	5'-TCTCGATGGCACTCTCTTCCG-3'	RT-PCR
RORβ	Forward	5'-TACGTGGTGGAGTTCGCCAAG-3'	RT-PCR
	Reverse	5'-CCCATGCAAGTTGCAGACTGC-3'	RT-PCR
LMO3	Forward	5'-GTTTGGTGTAACGGGAAACTGCG-3'	RT-PCR
	Reverse	5'-TCCTCGTAGTCTGTCTGGCAAAG-3'	RT-PCR
HMGA2	Forward	5'-ACATCAGCCCAGGGACAACCT-3'	RT-PCR
	Reverse	5'-CAAGAGTCCGCAGAGGAGGAT-3'	RT-PCR
EphrinB2	Forward	5'-TTCAGCCCTAACCTCTGGGGT-3'	RT-PCR
	Reverse	5'-AACCCAGGAGATTGTTCCCGG-3'	RT-PCR
mGAPDH	Forward	5'-CATCACCATCTTCCAGGAGC-3'	RT-PCR
	Reverse	5'-GCTGTAGCCGTATTCATTGTC-3'	RT-PCR
pri-miR-219-2	Forward	5'-TACGCAGCTCCCGAGATCTGGTG-3'	RT-PCR
	Reverse	5'-CAGCGTGGACCTCGTCTCTGTAG-3'	RT-PCR
pre-miR-219-2	Forward	5'-CTGATTGTCCAAACGCAATTCTTG-3'	RT-PCR
	Reverse	5'-CAGATGTCCAGCCACAATTCTC-3'	RT-PCR
PDGFRα	Forward	5'-GTGGCCTGGACGAACAGAGACT-3'	RT-PCR
	Reverse	5'-GGAACCTGTCTCGATGGCACTC-3'	RT-PCR
m TLX	Forward	5'-GGTTCAGACAGCTCCGATTAGAC-3'	RT-PCR
	Reverse	5'-TGGAGAGCGGCAATGGCGGCAGC-3'	RT-PCR
β-Actin	Forward	5'-CCGAGCGTGGCTACAGCTTC-3'	RT-PCR
	Reverse	5'-ACCTGGCCGTCAGGCAGCTC-3'	RT-PCR
hTLX	Forward	5'-CTAAGAGTGTGCCAGCCTTC-3'	RT-PCR
	Reverse	5'-TGTTAGCATCAACCGGAATGG-3'	RT-PCR
hGAPDH	Forward	5'-CCTGTTCGACAGTCAGCCG-3'	RT-PCR
	Reverse	5'-CGACCAAATCCGTTGACTC-3'	RT-PCR
